# Supplementary material for: AAV-delivered TurboRFP enables streamlined tracing and analysis of corticospinal tract sprouting
Source: Cell Rep Methods. 2026 Jul 31;6(9):101540. doi: 10.1016/j.crmeth.2026.101540 (PMC13615489; doi:10.1016/j.crmeth.2026.101540)
Supplement: Supplementary file 1 [file mmc1.pdf]

Cell Reports Methods, Volume *6*

Supplemental information

AAV-delivered TurboRFP enables streamlined tracing and analysis of corticospinal tract sprouting

Marc Hernaiz-Llorens, Camilo J. Londoño, Ellen Wang, Felicia Cai, Junmi M. Saikia, Carmine L. Chavez-Martinez, Hugo J. Kim, Chimuanya K. Agba, and Binhai Zheng

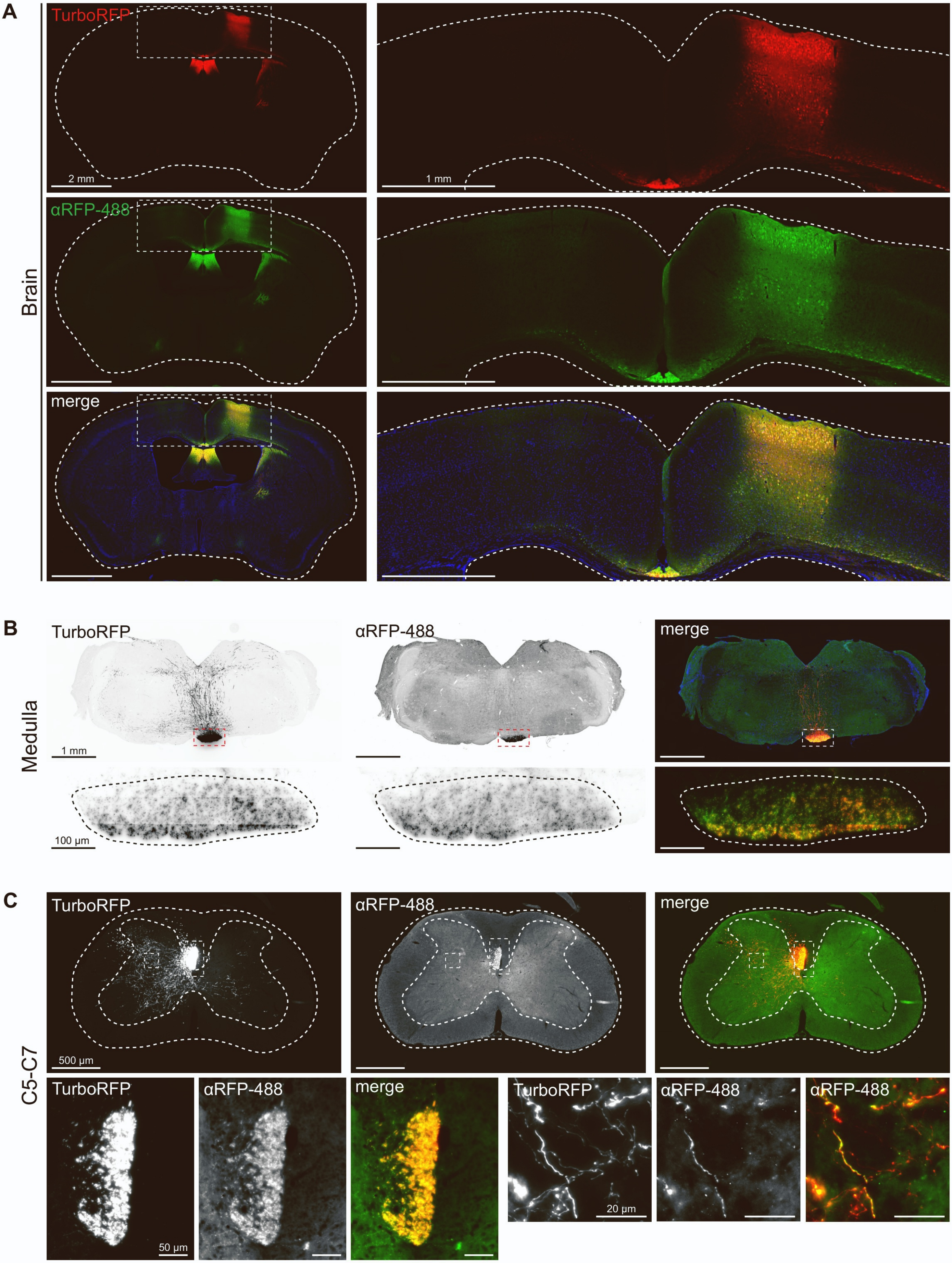

Figure S1. Immunodetection of TurboRFP tracing along the CST. Related to Figure 1, 2 and 4. (A) Coronal brain sections showing TurboRFP labeling (red) and corresponding αRFP-488 immunostaining (green). Merged images confirm largely overlapping signals. The same color scheme applies for merged images below. **(B)** Transversal medulla sections showing TurboRFP-labeled CST axons and overlapping αRFP-488 immunostaining signal. **(C)** Transversal cervical spinal cord sections (C5-C7) showing CST and collateral fibers labeled with TurboRFP. Immunolabeling with αRFP-488 showed clear signal within the main CST but did not improve visualization of fine CST collaterals or varicosities in the cervical spinal cord and introduced substantial background signal under our experimental conditions. Higher power insets show details of native TurboRFP and immunodetected TurboRFP signals in the main CST and collateral fibers.

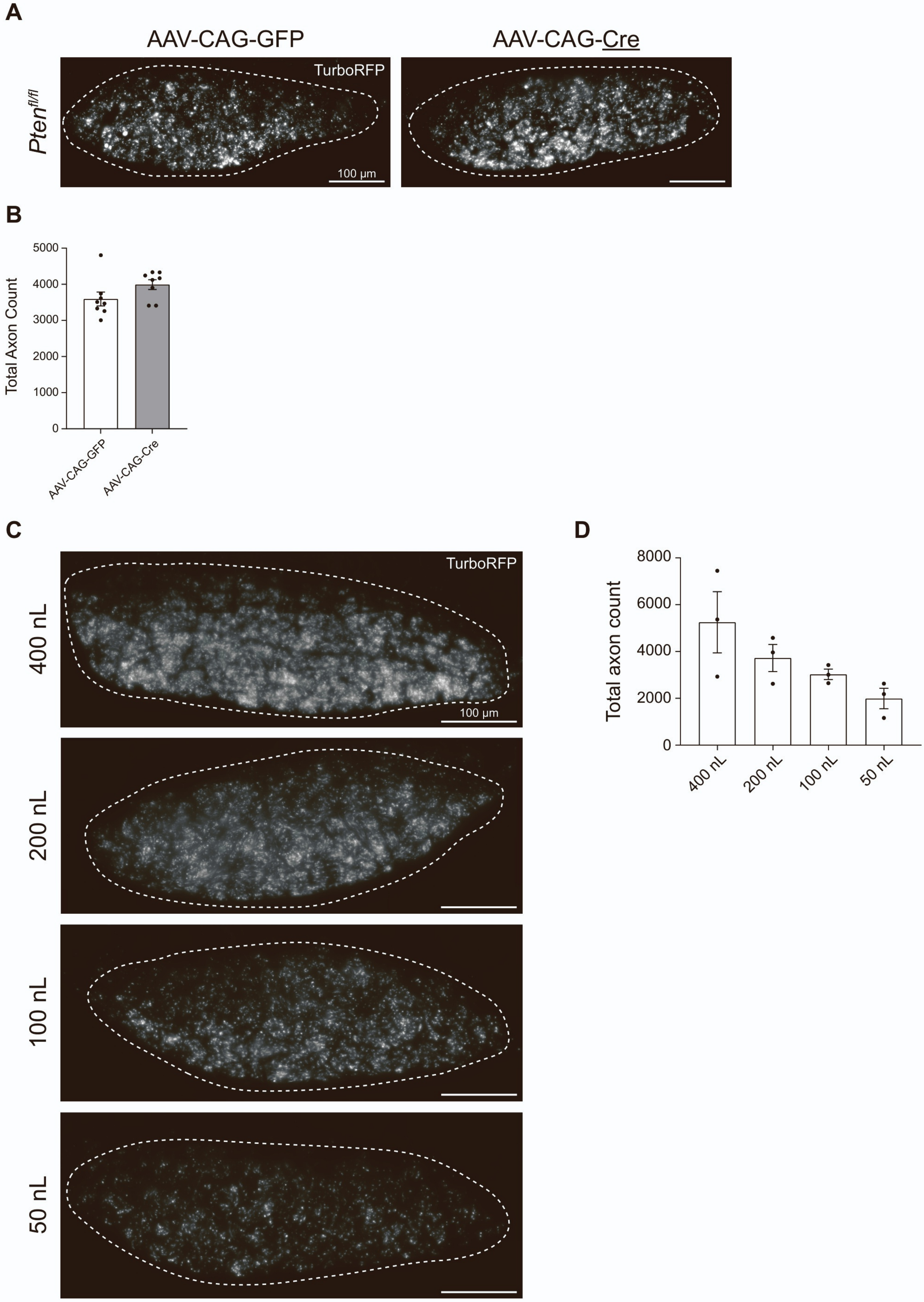

Figure S2. Effects of Pten deletion and AAV1-hSyn-TurboRFP injection volume on total CST axon counts in the medulla. Related to Figures 2, 3, and 4. (A) Representative transverse sections of TurboRFP traced axons in the CST at the medullary pyramid level from Ptenfl/fl mice injected with either AAV2-CAG-GFP (control) or AAV2-CAG-Cre, showing comparable TurboRFP-labeled axons. **(B)** Quantification of total TurboRFP-positive axons at the medullary level showing no significant difference between AAV-CAG-GFP and AAV-CAG-Cre injected groups. Results are presented as mean ± SEM (n = 8 animals per experimental group, unpaired t-test). **(C)** Representative transverse sections of TurboRFP traced axons in the CST at the medullary pyramid level injected with different volumes of AAV1-hSyn-TurboRFP (400 nL, 200 nL, 100 nL or 50 nL per injection site) into the cortex of wild-type animals. **(D)** Quantification of total CST axon counts across injection volumes. Although no statistically significant differences were detected across the different injection volumes, there was a trend for a modest decline in labeling efficiency as injection volume reduced. Results are presented as mean ± SEM (n = 3 animals per experimental group, 3 medulla sections analyzed per animal, one-way ANOVA with post-hoc Tukey's multiple comparison test).

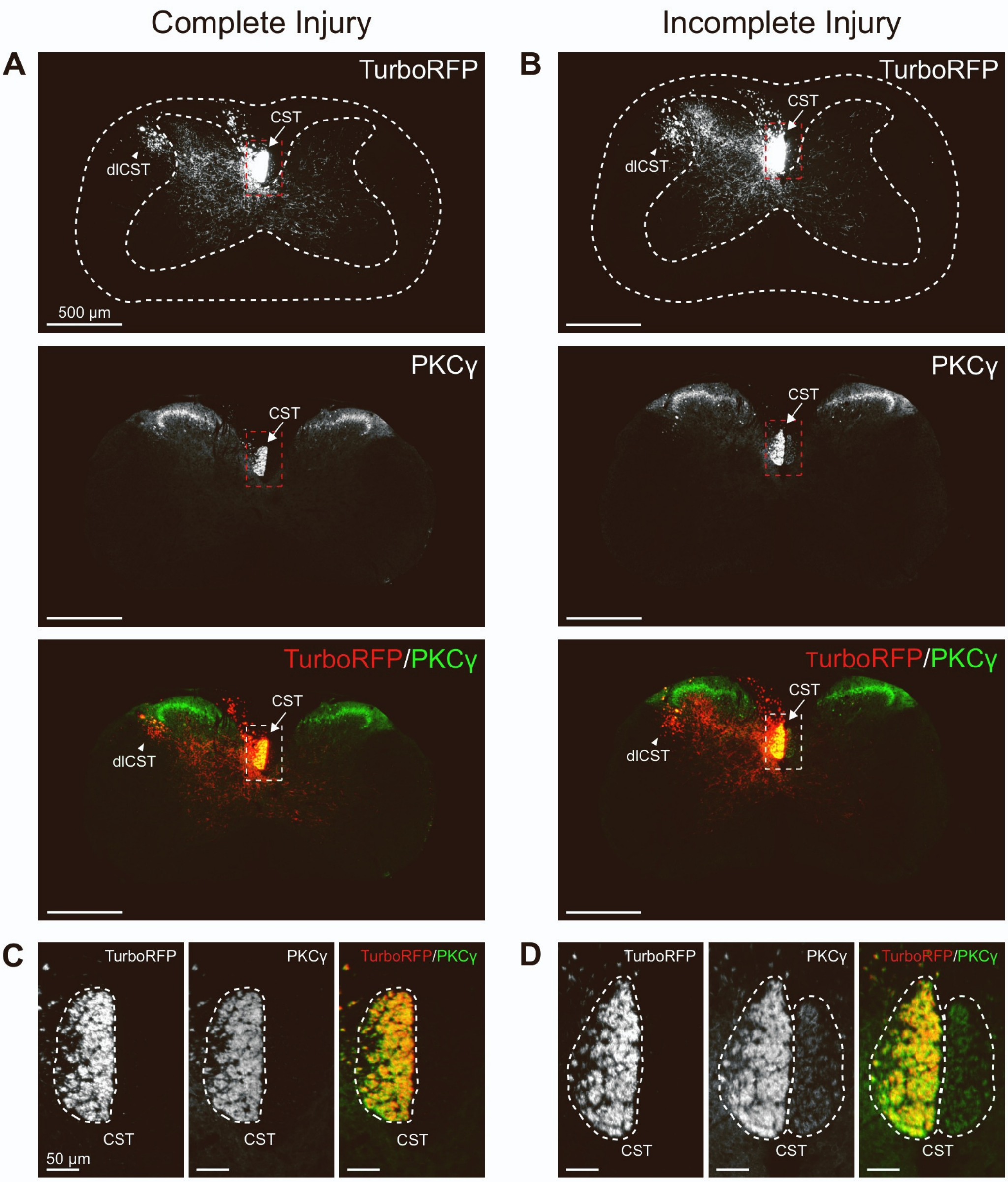

Figure S3. Verification of the pyramidotomy lesion completeness by PKCγ detection. Related to Figure 4. (A) Representative transverse sections of the cervical spinal cord (C5-C7) from an animal with a complete injury, showing TurboRFP labeling of the intact CST, collateral projections and the dorsolateral CST (dlCST) and loss of PKCγ immunoreactivity within the lesioned CST. **(B)** Example of an incomplete injury, showing residual PKCγ immunoreactivity within the partially injured CST. (C, D) Higher-magnification views of the CST region in complete **(C)** and incomplete **(D)** injuries, illustrating the absence or persistence of PKCγ signal, respectively.

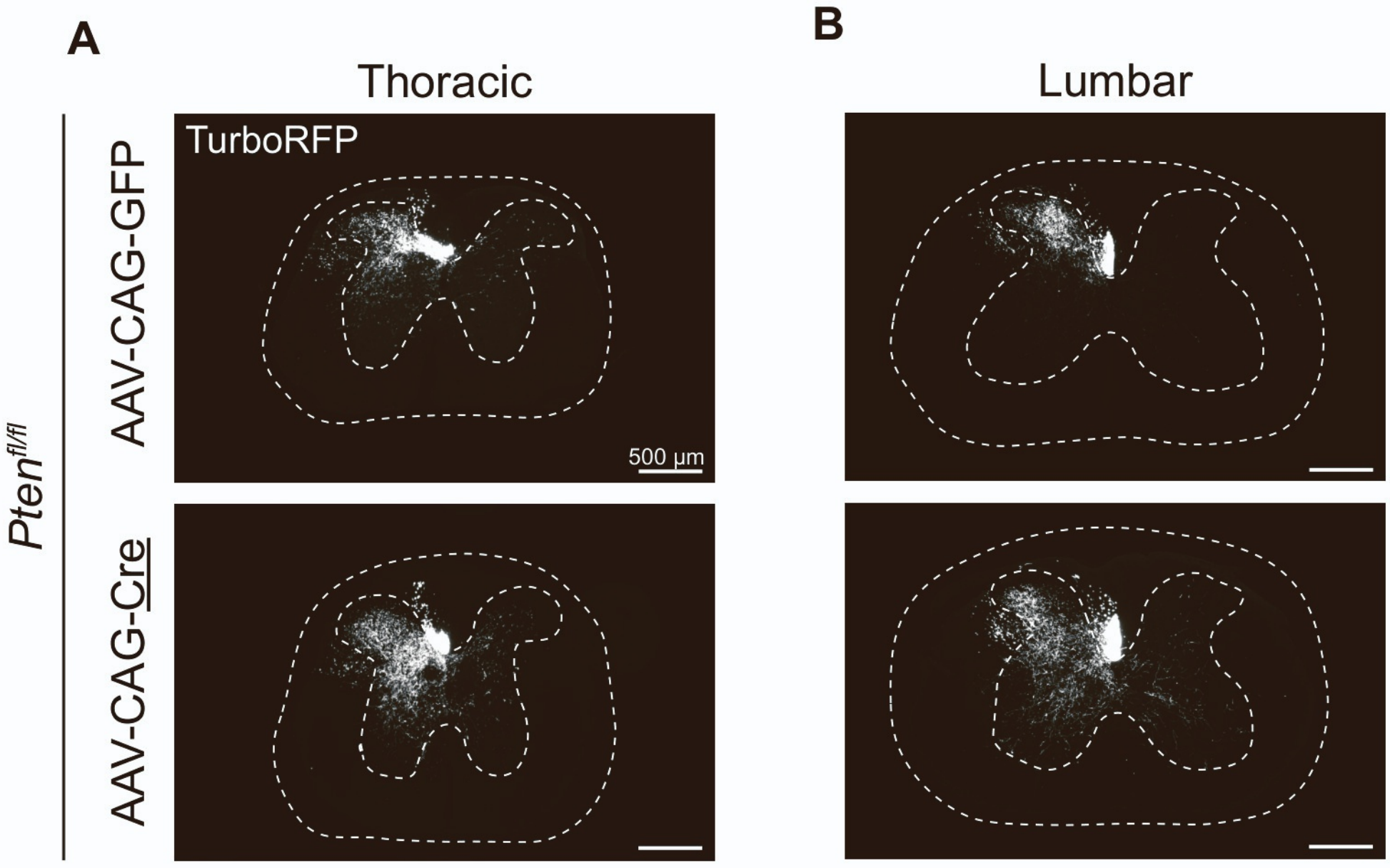

Figure S4. TurboRFP labeling of CST axons extends to thoracic and lumbar spinal cord levels. Related to Figure 4. (A) Representative transverse sections showing TurboRFP-labeled corticospinal tract axons in thoracic segments of the spinal cord from Ptenfl/fl mice injected with AAV-CAG-GFP (control) or AAV-CAG-Cre (Pten deletion) together with AAV1-hSyn-TurboRFP. **(B)** Representative transverse sections showing TurboRFP-labeled corticospinal tract axons in lumbar segments of the spinal cord from Ptenfl/fl mice injected with AAV-CAG-GFP (control) or AAV-CAG-Cre (Pten deletion) together with AAV1-hSyn-TurboRFP.

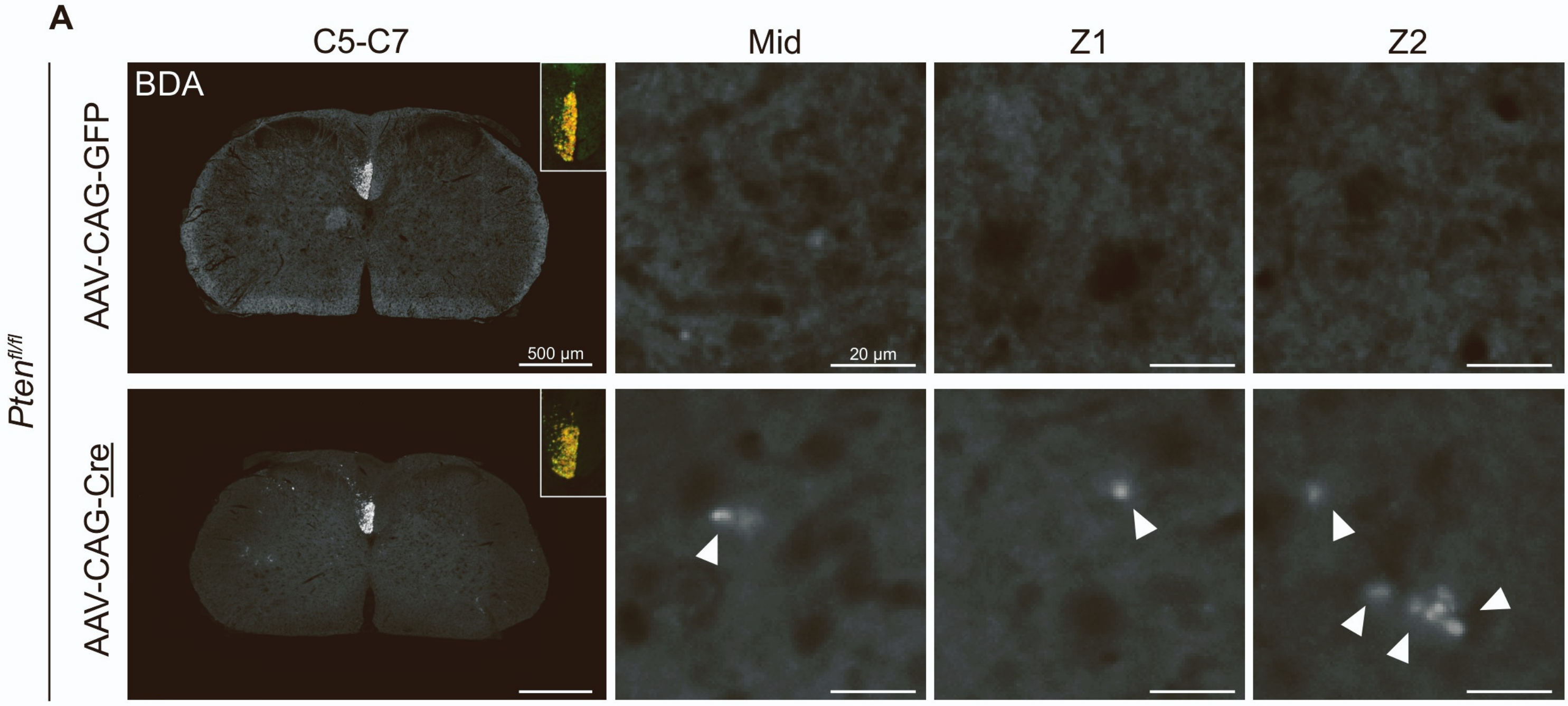

Figure S5. Detection of sparse BDA-labeled CST collaterals after unilateral pyramidotomy. Related to Figure 4. (A) Representative transverse sections of the C5–C7 cervical spinal cord from Ptenfl/fl mice injected with AAV-CAG-GFP or AAV-CAG-Cre and labeled with BDA. Higher-magnification views of the Mid, Z1, and Z2 regions illustrate the sparse and minimally detectable BDA-positive CST collateral fibers (arrowheads) observed after Pten deletion. BDA was detected using fluorescently conjugated streptavidin without signal amplification. Color insets show BDA labeling (green) and native TurboRFP signal (red) within the main CST.

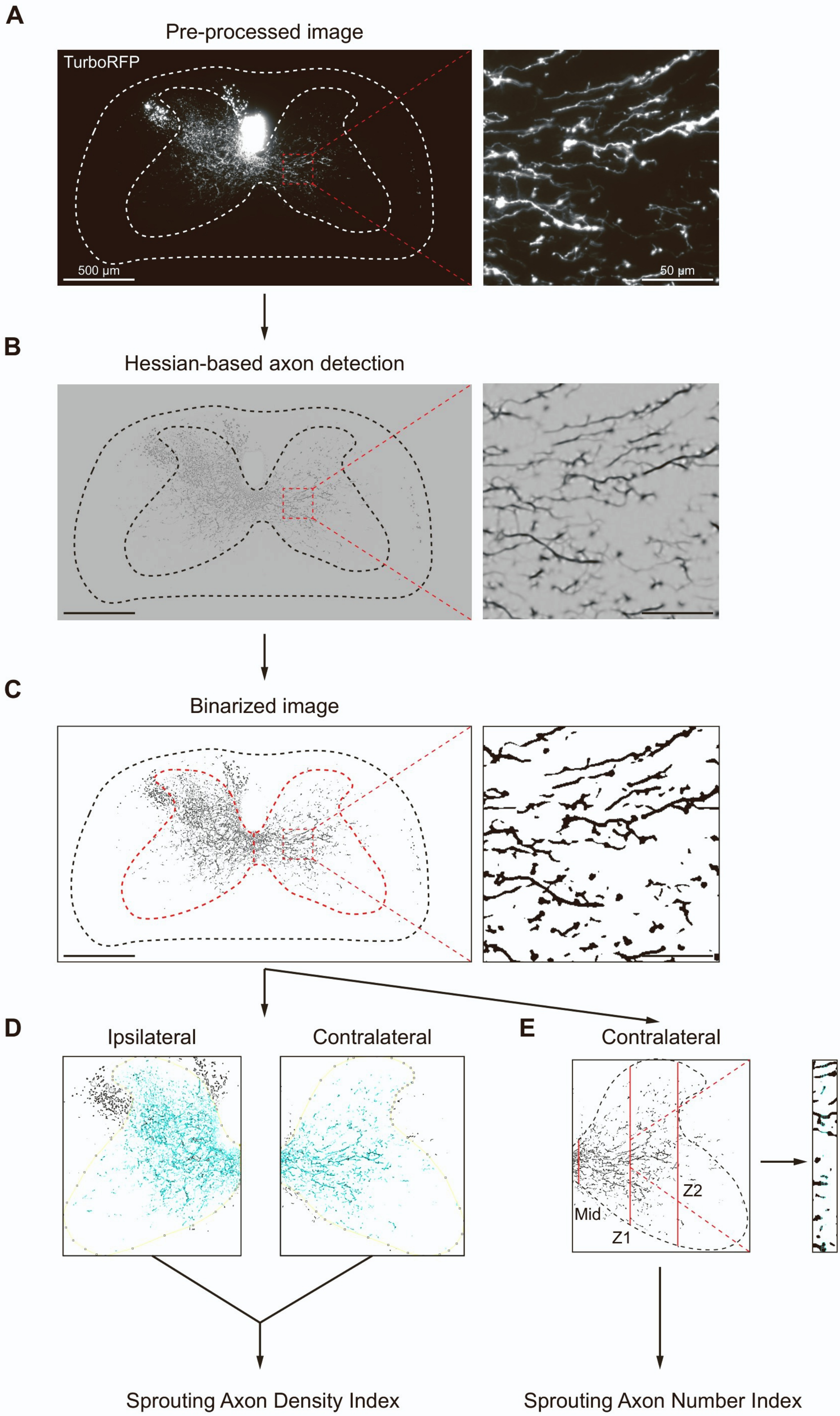

Figure S6. Semi-automated FeatureJ-based pipeline for CST sprouting analysis of CST sprouting using FeatureJ. Related to Figure 4. (A) Pre-processed TurboRFP image of a transverse cervical spinal cord section showing collateral and sprouting fibers. **(B)** Image resulting after application of smallest Hessian value, with a smoothing scale of 2, with FeatureJ plugin. **(C)** Binarized image used for subsequent analysis. **(D)** Corresponding binarized sections of the ipsilateral and contralateral gray matter used to calculate the Sprouting Axon Density Index. **(E)** Definition of mediolateral zones (Mid, Z1, Z2) within the contralateral gray matter for calculating the sprouting axon number index, representing the number of discrete axons crossing each zone.
