# Supplementary material for: AAV-delivered TurboRFP enables streamlined tracing and analysis of corticospinal tract sprouting
Source: Cell Rep Methods. 2026 Jul 31;6(9):101540. doi: 10.1016/j.crmeth.2026.101540 (PMC13615489; doi:10.1016/j.crmeth.2026.101540)
Supplement: Supplementary file 2 [file mmc2.pdf]

Cell Reports
Methods

Report

AAV-delivered TurboRFP enables streamlined tracing and analysis of corticospinal tract sprouting

Graphical abstract

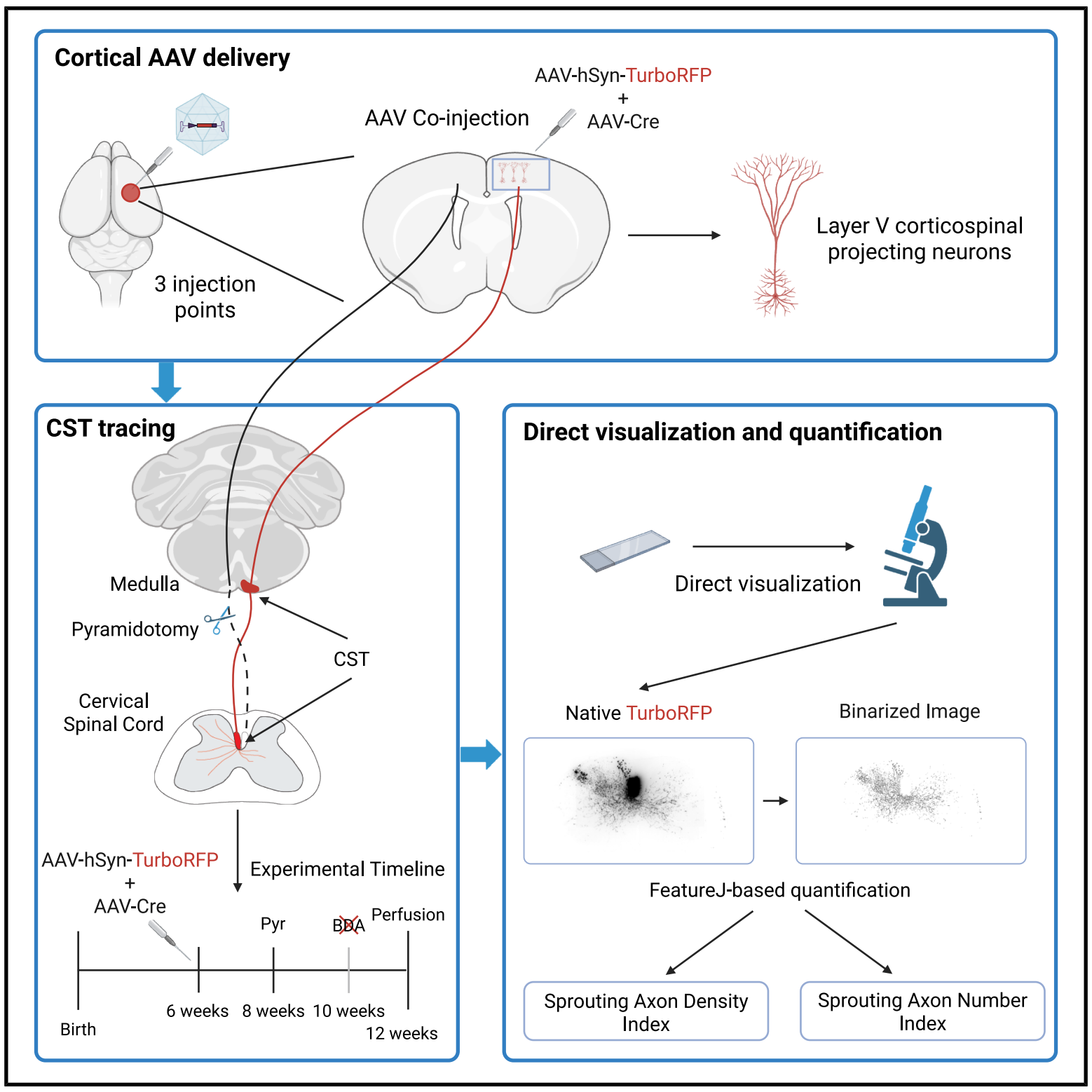

Authors

Marc Hernaiz-Llorens,
Camilo J. Londoño, Ellen Wang, ...,
Hugo J. Kim, Chimuanya K. Agba,
Binhai Zheng

Correspondence

bizheng@health.ucsd.edu

In brief

Hernaiz-Llorens et al. establish an AAV1-hSyn-TurboRFP workflow for streamlined corticospinal tract sprouting analysis. The method enables direct visualization of fine CST axons without secondary immunostaining, supports combined tracing and genetic manipulation, and provides reproducible quantification of injury-induced corticospinal remodeling.

Highlights

- AAV1-hSyn-TurboRFP enables simple and robust labeling of corticospinal axons
- Minimal tissue processing reduces variability and improves reproducibility
- Standardized workflow streamlines reliable quantification of CST sprouting
- Co-injection with other AAVs enables combinatorial manipulation

Hernaiz-Llorens et al., 2026, Cell Reports Methods 6, 101540
September 21, 2026 © 2026 The Author(s). Published by Elsevier Inc.
https://doi.org/10.1016/j.crmeth.2026.101540

CellPress

Cell Reports Methods

CellPress
OPEN ACCESS

Report

AAV-delivered TurboRFP enables streamlined tracing and analysis of corticospinal tract sprouting

Marc Hernaiz-Llorens,[1] Camilo J. Londoño,[1,2] Ellen Wang,[1,2] Felicia Cai,[1] Junmi M. Saikia,[1,2] Carmine L. Chavez-Martinez,[1,3] Hugo J. Kim,[1] Chimuanya K. Agba,[1,2] and Binhai Zheng[1,4,5,*]
[1]Department of Neurosciences, School of Medicine, University of California San Diego, La Jolla, CA, USA
[2]Neurosciences Graduate Program, University of California San Diego, La Jolla, CA, USA
[3]Biological Sciences PhD Program, University of California San Diego, La Jolla, CA, USA
[4]VA San Diego Research Service, San Diego, CA, USA
[5]Lead contact
*Correspondence: bizheng@health.ucsd.edu
https://doi.org/10.1016/j.crmeth.2026.101540

MOTIVATION Studies of corticospinal tract (CST) axon repair after CNS injury rely on efficient and reliable methods for robust axon tracing. The commonly used chemical tracer biotinylated dextran amine (BDA) has limited labeling sensitivity, requires additional surgical procedures, and entails labor-intensive and time-consuming tissue processing with substantial variability. State-of-the-art viral tracers can robustly label CST axons but often require extensive tissue processing and signal amplification. A simple, efficient, and reliable method that enables direct visualization of CST axons without immunostaining or signal amplification would lower technical barriers to axon repair research and improve reproducibility across laboratories.

SUMMARY

Quantitative analysis of corticospinal tract (CST) sprouting after injury requires reliable labeling of long-range axons and fine collateral branches. Conventional biotinylated dextran amine (BDA) tracing has limited sensitivity and requires additional surgeries, while some viral-based approaches, although robust, rely on extensive tissue processing and signal amplification. Here, we describe a streamlined adeno-associated virus (AAV)-based workflow for CST sprouting analysis using TurboRFP that enables robust labeling of descending CST axons, including sprouting fibers after unilateral pyramidotomy. This approach allows direct visualization of fine CST axons without immunostaining or signal amplification, simplifying tissue processing and reducing experimental variability. Using a standardized workflow, we enable consistent CST labeling and reproducible quantification of CST remodeling. In addition, compatibility with co-delivery of other AAVs enables simultaneous circuit tracing and genetic manipulation within the same neuronal population. This workflow provides a practical platform that lowers technical barriers to axon repair research and potentially improves reproducibility across laboratories.

INTRODUCTION

Quantitative analysis of long-range axonal projections requires reliable labeling of projection neurons and their axons. The corticospinal tract (CST), a major descending motor pathway, is widely used to study axonal plasticity and regeneration after spinal cord injury (SCI).[1,2] Because CST axons extend over long distances and exhibit limited spontaneous regeneration, this system provides a stringent and well-established platform for evaluating axonal growth and remodeling after injury.[3,4]

Biotinylated dextran amine (BDA), a classical chemical anterograde tracer, has been extensively used to label CST axons.[5,6] BDA tracing has supported major advances in CST biology, including enhanced regeneration following *Pten* deletion[7] and extensive spontaneous corticospinal plasticity after primate SCI.[8] Detection of BDA-labeled axons typically requires signal amplification, which, historically, was performed using chromogenic methods such as diaminobenzidine (DAB) and, more recently, involves fluorescent approaches, adding processing steps that can increase variability.

Adeno-associated virus (AAV)-based methods have emerged as versatile tools for neural circuit tracing and manipulation, enabling targeted labeling and genetic access to specific neuronal populations.[9,10] These approaches have been used to label CST

This is an open access article under the CC BY license (http://creativecommons.org/licenses/by/4.0/).

axons and identify regulators of CST regeneration and remodeling.[11,12] Efforts to improve AAV-based axon tracing have focused on optimizing viral serotypes and promoters to enhance labeling efficiency.[13,14]

However, robust detection of fine axons often still relies on immunostaining or signal amplification, which can be labor intensive and variable across experiments. Although AAV-mediated fluorescent tracing is widely used and prior studies have compared AAV-based labeling with BDA in the brain, fewer studies have addressed practical workflows for detecting sparse CST collateral and sprouting axons within the spinal cord after injury.

Here, we evaluate an AAV1-hSyn-TurboRFP-based workflow for CST sprouting analysis after unilateral pyramidotomy, in comparison with conventional BDA tracing. Rather than introducing a new class of axonal tracer, this study establishes a simplified and reproducible pipeline for detecting and quantifying CST remodeling after injury. Our results demonstrate that AAV-TurboRFP enables robust CST labeling without immunostaining or signal amplification, providing a practical platform for quantitative analysis of axonal sprouting.

RESULTS

AAV1-hSyn-TurboRFP robustly transduces CST neurons in the sensorimotor cortex

AAVs are widely used gene delivery vectors characterized by broad but tunable neuronal tropism, low pathogenicity, and minimal immunogenicity.[15] Different AAV serotypes exhibit marked differences in the transduction efficiency, cell type specificity, and axonal transport properties.[16–18] Among these, AAV1 has been shown to be particularly effective for neuronal transduction and is among the most efficient serotypes for labeling CST neurons and their axons.[13,14,19] In addition, the human synapsin (hSyn) promoter provides an optimal balance of neuronal specificity and expression efficiency for CST labeling.[14,20]

Based on these considerations, we used an AAV1-hSyn-TurboRFP construct (Addgene viral prep #105552-AAV1; RRID: Addgene_105552), in which the hSyn promoter drives expression of the dimeric red fluorescent protein TurboRFP.[21] TurboRFP exhibits high brightness, rapid maturation, and the excitation/emission peak at 553/574 nm, while complementing commonly used GFP-based vectors. A schematic of the vector construct is shown in Figure 1A.

To label cervical-projecting corticospinal neurons (CSNs), we injected AAV1-hSyn-TurboRFP into the forelimb sensorimotor cortex of 6-week-old mice and collected tissue 6 weeks later (Figures 1B and 1C). Viral particles were delivered across three cortical sites to maximize consistency and coverage of the forelimb CSN territory (Figures 1C and 1D; see STAR Methods). Robust TurboRFP fluorescence was observed without the need for immunostaining or signal amplification across the motor cortex at all injection sites (Figure 1D). Labeled neurons were distributed predominantly in cortical layers II/III and V (Figure 1E), reflecting labeling of corticocortical neurons and layer V projection neurons, including CSNs. Labeled CST axons could be readily identified along their trajectory, including fiber bundles within the corpus callosum (cc) and internal capsule (ic) (Figures 1F and 1G). These axons converge at the medullary pyramids (Py) and decussate (Dec) and can be continuously traced caudally along the CST toward the spinal cord (Figure 1H).[22] Fine axonal processes could be clearly resolved, and the pyramidal decussation was sharply delineated, highlighting the sensitivity of AAV1-mediated fluorescent tracing for resolving corticospinal projections (Figure 1H).

AAV1-hSyn-TurboRFP provides more efficient cortical labeling than BDA and enables standardized CST axon quantification at the level of the pyramidal decussation

BDA is a widely used anterograde tracer for mapping neural circuits and assessing axonal regeneration and sprouting, typically detected using avidin-biotin complex methods or fluorescently conjugated streptavidin.[5] To compare viral and conventional tracing strategies, the mice received AAV1-hSyn-TurboRFP injections followed 4 weeks later by BDA injections at matching cortical coordinates; tissues were collected 2 weeks after BDA injection (Figures 2A and 2B). TurboRFP was detected by native fluorescence, whereas BDA was detected using fluorescently labeled streptavidin without amplification, enabling within-animal comparison under minimally processed conditions.

AAV1-hSyn-TurboRFP injections produced robust and widespread fluorescence across cortical layers without the need for secondary staining or signal amplification (Figure 2C). In matched sections, TurboRFP labeled more CSNs and covered a larger cortical area than BDA (Figures 2D and 2E). Immunodetection of RFP (detected with Alexa Fluor 488, in green) produced signals that largely overlapped with native TurboRFP fluorescence (in red) across the CNS (brain, medulla, and cervical spinal cord) but did not improve visualization of fine CST collaterals or varicosities in the cervical spinal cord and introduced substantial background signal under our experimental conditions (Figure S1); therefore, this approach was not pursued further. Quantitative analyses confirmed that both the number of labeled neurons (~5-fold increase) and the cortical area occupied by labeled somata (~7-fold increase) were significantly greater with TurboRFP than with BDA (Figure 2F).

To establish a reliable normalization metric across animals, we quantified labeled CST axons at the level of the Py for both tracers (Figures 2G and 2H). TurboRFP consistently yielded higher axon counts (~8-fold increase) than BDA (Figures 2I and 2J). These standardized axon counts served as an internal reference for downstream analyses of spinal projections in both injured and uninjured animals.

Collectively, these results demonstrated that AAV1-hSyn-TurboRFP provides a simpler, more efficient, and more reproducible approach for labeling corticospinal projections than BDA, enabling robust within-animal normalization and improved quantification across experiments.

TurboRFP-labeled CSNs show robust pS6 upregulation following Cre-mediated *Pten* deletion

The study of axonal sprouting after pyramidotomy commonly employs *Pten* deletion as a positive control, as this manipulation has been well established to enhance axon regeneration and sprouting after injury.[7,23] Conditional deletion of *Pten*, a negative regulator of the mTOR pathway, in CSNs leads to robust activation of mTORC1 signaling, which can be detected by increased

CellPress
OPEN ACCESS

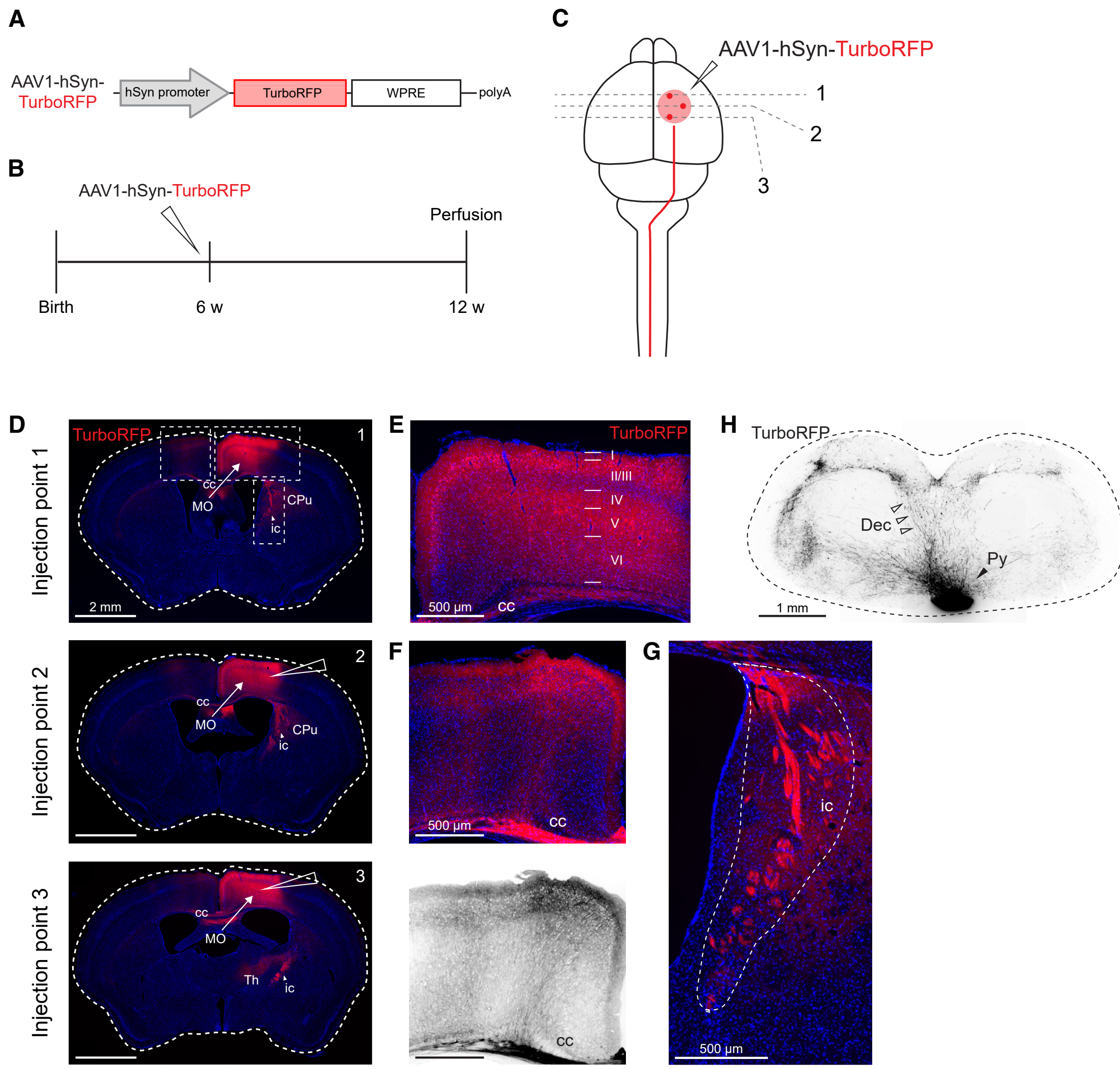

Figure 1. Experimental design and TurboRFP expression in the mouse brain
(A) AAV1-hSyn-TurboRFP vector design.
(B) Experimental timeline for cortical AAV injection and tissue collection.
(C) Schematic of the AAV1-hSyn-TurboRFP injection sites in the sensorimotor cortex.
(D) TurboRFP expression across three cortical injection sites.
(E) TurboRFP-labeled neurons in cortical layers II/III and V.
(F and G) TurboRFP-labeled axons projecting through the corpus callosum (cc) (F) and internal capsule (ic) (G).
(H) TurboRFP-labeled corticospinal axons descending through the medullary pyramid (Py) and pyramidal decussation (Dec).

pS6 levels.[7,24] To induce *Pten* deletion, we injected AAV2-CAG-Cre into the forelimb region of the sensorimotor cortex of *Pten*$^{fl/fl}$ mice, with AAV2-CAG-GFP as a control (Figures 3A and 3B). Cre- or GFP-expressing vectors were co-injected with AAV1-hSyn-TurboRFP. Four weeks later, BDA was injected at the same cortical sites, and tissues were collected two weeks later for histological analysis (Figure 3B).

To enable co-injection, the volume of AAV1-hSyn-TurboRFP was optimized by testing multiple volumes (400, 200, 100, and 50 nL) and quantifying labeled CST axons at the level of the Py. Although no statistically significant differences were observed, a clear trend toward increased labeling with higher volumes was noted; 200 nL was, therefore, selected as a compromise between the labeling efficiency and injection volume for co-injections (Figure S2).

As expected, pS6 immunoreactivity was strongly upregulated in the Cre-injected hemispheres (Figure 3C), and quantitative analysis confirmed increased pS6 signal relative to the contralateral side, consistent with activation of the mTORC1 pathway in CSNs (Figure 3D). Notably, pS6 upregulation closely overlapped with TurboRFP-labeled cortical regions and neurons, indicating efficient co-targeting of the same neuronal population for both

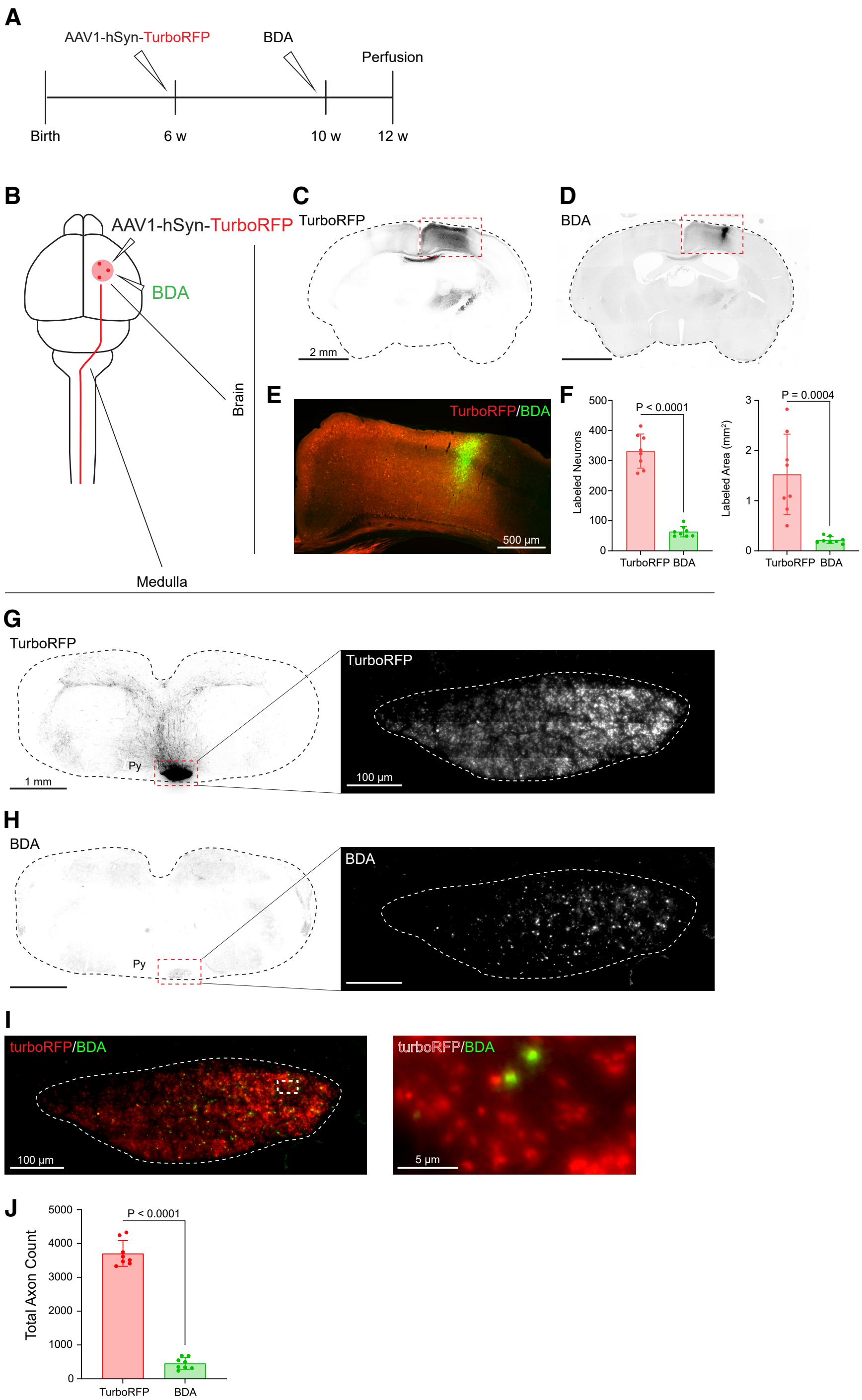

Figure 2. Comparison of labeling efficiency between AAV-mediated TurboRFP expression and BDA

(A) Experimental timeline for AAV1-hSyn-TurboRFP and BDA injections.

(B) Schematic of the cortical injection sites.

(C and D) Coronal brain sections showing TurboRFP (C) and BDA (D) labeling.

(E) High-magnification and merged images showing TurboRFP and BDA labeling in the cortex.

(F) Quantification of labeled neurons and cortical labeling area per injection site. Data are mean ± SEM; $n = 8$ animals, 3 sections per animal; paired t test.

(G and H) Transverse medulla sections showing TurboRFP-labeled (G) and BDA-labeled (H) CST axons.

(I) Merged TurboRFP and BDA labeling at the medullary level.

(J) Quantification of total labeled CST axons in the medulla. Data are mean ± SEM; $n = 8$ animals per group, 3 sections per animal; paired t test.

genetic manipulation and neuronal labeling (Figure 3E). In contrast, BDA labeled a smaller subset of cortical neurons with limited overlap with pS6-positive cells. These findings demonstrated that Cre-mediated *Pten* deletion and TurboRFP labeling target the same and broader population of CSNs, supporting this approach as a unified platform for simultaneous genetic manipulation and axonal tracing.

TurboRFP reliably detects enhanced CST sprouting in *Pten*-deleted mice after unilateral pyramidotomy

To evaluate the ability of TurboRFP to trace and detect injury-induced CST plasticity, we applied our tracing strategy to a unilateral pyramidotomy model, a well-established paradigm for assessing axon sprouting from the spared CST.[25] In this model, one Py is transected, leaving the contralateral CST intact to serve as a substrate for compensatory sprouting toward the denervated cervical spinal cord (Figure 4B). All animals were assessed for lesion completeness by immunostaining for PKCγ in the cervical spinal cord; animals exhibiting residual CST immunoreactivity were excluded (Figure S3).

Conditional *Pten* deletion was induced in layer V CSNs of $Pten^{fl/fl}$ mice via cortical injection of AAV2-CAG-Cre, whereas control animals received AAV2-CAG-GFP. Consistent with previous studies, *Pten* loss robustly enhances CST regeneration and axon sprouting after injury.[7] To directly compare the tracing performance, both TurboRFP and BDA were used to label CST axons within the same animals (Figure 4C). Importantly, *Pten* deletion did not alter the total number of labeled axons at the level of the Py, indicating comparable labeling efficiency across groups (Figure S2).

As expected, only limited spontaneous sprouting was observed in control animals, whereas AAV-Cre-injected mice exhibited a marked increase in CST collateralization within the

CellPress
OPEN ACCESS

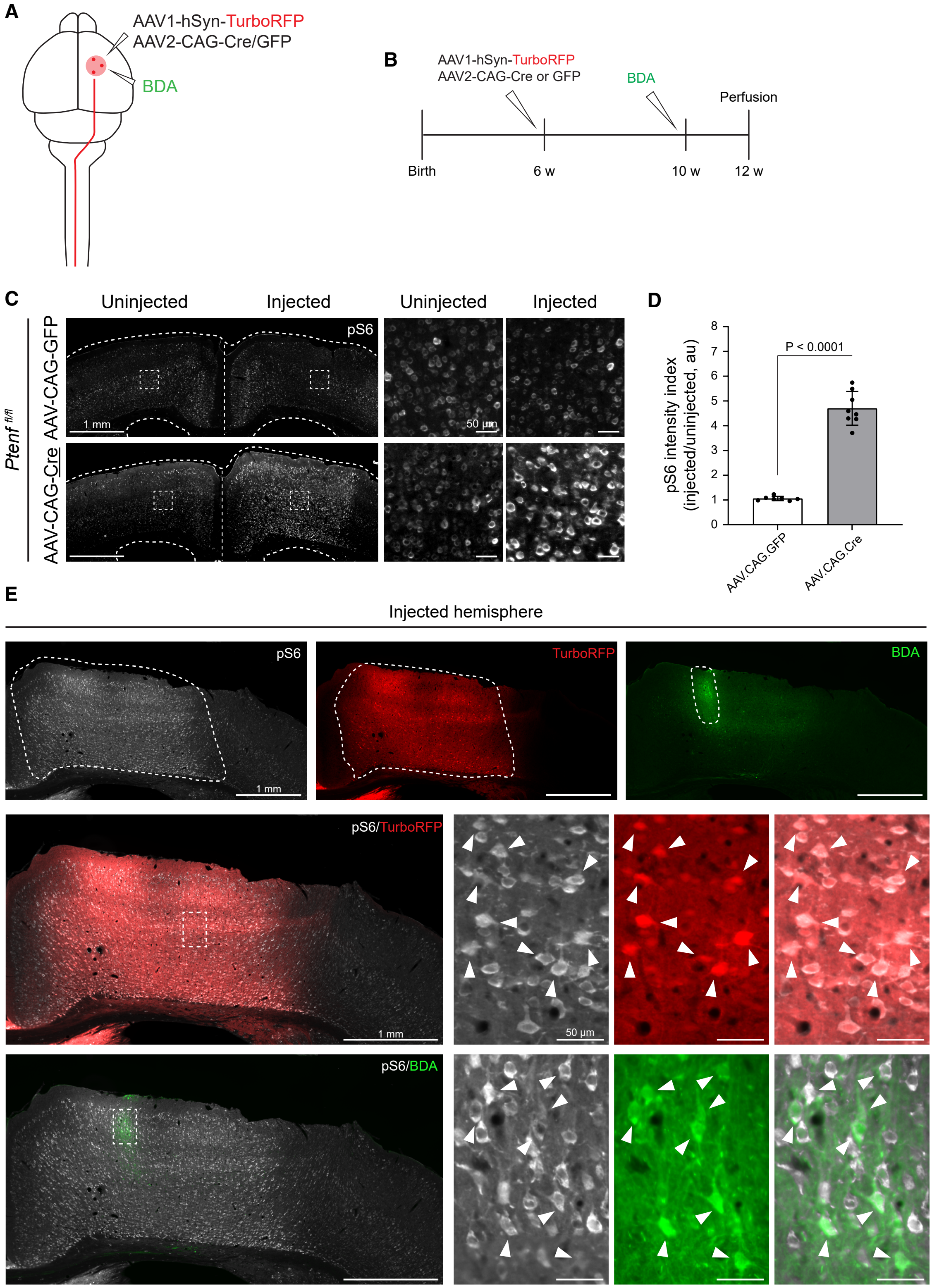

Figure 3. TurboRFP labeling and pS6 upregulation after AAV-Cre-mediated *Pten* deletion

(A) Schematic of cortical co-injection of AAV1-hSyn-TurboRFP with AAV2-CAG-GFP or AAV2-CAG-Cre in $Pten^{fl/fl}$ mice, followed by BDA injection.

(B) Experimental timeline.

(legend continued on next page)

denervated side of the cervical spinal cord (Figure 4A). TurboRFP labeling produced strong fluorescence signals that clearly delineated fine axonal branches, enabling robust visualization of sprouting axons.

Although our cortical injections were targeted to the forelimb sensorimotor cortex to preferentially label cervical-projecting CST neurons, TurboRFP-positive CST axons and collateral fibers were also detectable at thoracic and lumbar spinal cord levels without immunostaining or signal amplification (Figures S4A and S4B). This likely reflected the spread of AAV around the injection site, together with overlapping coordinates between the forelimb and hindlimb cortical areas.[23,26] As expected, the labeling intensity was reduced at more caudal spinal cord levels relative to the cervical cord. These data indicate that TurboRFP can also be effective for tracing distal CST axons and collateral sprouting.

In contrast, BDA signal, detected using fluorescently conjugated streptavidin to allow a more direct comparison with native TurboRFP fluorescence, did not reliably label collateral and sprouting axons and was largely restricted to the main CST tract. Only sparse BDA$^+$ fibers were observed in *Pten*-deleted animals (Figure S5, arrowheads) and appeared more abundant than in controls, but these were not quantified due to the low overall signal intensity. High-magnification views (Mid, Z1, and Z2) revealed numerous TurboRFP-labeled axons extending throughout the denervated gray matter (Figure 4A).

Quantitative analysis of TurboRFP images revealed a significant increase in both the sprouting axon density index (Figure 4D) and the sprouting axon number index across all mediolateral regions examined (Figure 4E). The sprouting axon density index was defined as the CST axon density in the gray matter of the contralateral (denervated) side normalized to the ipsilateral (intact) side, whereas the sprouting axon number index represents the number of CST axons within three predefined regions (Mid, Z1, and Z2), normalized to the total CST axon counts in the medulla. Together, these results demonstrated that TurboRFP tracing enables rapid, high-contrast, and quantitative visualization of CST axon sprouting, with improved sensitivity and reliability compared with conventional BDA labeling. Notably, because *Pten* deletion may also enhance sprouting on the intact side, normalization to the ipsilateral (intact) side (as in sprouting axon density index) underestimates the overall magnitude of the sprouting effect.

FeatureJ-based quantification enables standardized analysis of CST sprouting

To enable objective and efficient quantification of CST sprouting after unilateral pyramidotomy, we implemented a semi-automated image-processing workflow in Fiji, using the FeatureJ plugin, which enhances the detection of tubular structures such as axons and facilitates sensitive identification of CST collaterals and sprouting fibers.[27] Images of TurboRFP-labeled CST terminals in the cervical spinal cord were first subjected to background subtraction (Figure S6A), followed by application of the FeatureJ smallest Hessian eigenvalue filter (smoothing scale = 2) to enhance axon-like structures, while minimizing background signal (Figure S6B). The smoothing scale was empirically optimized using representative sections and then applied consistently across sections and groups to reduce user-dependent variability. Overexposed signals within the main CST were suppressed during this process, improving selective detection of collateral and sprouting axons (Figure S6C). Processed images were then thresholded and binarized to generate segmented masks for quantitative analysis (Figure S6D).

From these binary images, two complementary metrics were derived. The sprouting axon density index was defined as the ratio of total axonal signal within the contralateral (denervated) gray matter relative to the ipsilateral (intact) side (Figure S6D). The sprouting axon number index quantified axonal intersections within a narrowly defined, line-shaped region of interest (ROI) positioned along the denervated gray matter (Mid, Z1, and Z2), normalized to total CST axon counts in the medulla (Figure S6E). This semi-automated pipeline enables reproducible quantification of CST sprouting across animals and conditions.

DISCUSSION

In this study, we show that AAV1-hSyn-TurboRFP enables robust CST labeling and sprouting analysis after unilateral pyramidotomy without immunostaining or signal amplification. Combined with a standardized quantification workflow, this approach reduces tissue-processing complexity and supports reproducible assessment of CST remodeling after injury.

Robust labeling of CSNs and CST axons using native fluorescence

Vector injection into forelimb-targeting motor cortex robustly labeled layer V cortical neurons including CSNs, without the need for additional detection steps. The strong native fluorescence signal is in line with other AAV-based tracers, such as in a study with AAV-EGFP.[11] However, robust labeling of CST axons, including fine collaterals, with AAV-mediated fluorescent protein expression often requires immunostaining, such as in the case of AAV-ChR2-mCherry and AAV-ChR2-YFP,[12] or a GFP transgene expressed under the promoter of mu-crystallin (crym-GFP).[28]

Notably, TurboRFP has been employed in a number of studies to visualize neuronal processes *in vitro*[29–31] and long-range axons in the brain and spinal cord,[32–38] which underscores its reliability in tracing pathways that span large CNS distances. Specifically, AAV1-TurboRFP has been used to label regenerating CST axons but required immunostaining.[36] It has also been used in an intersectional labeling strategy to identify specific CST populations, detected by native fluorescence.[34] Together,

(C) Representative coronal sections showing pS6 immunoreactivity in control and *Pten*-deleted hemispheres.

(D) Quantification of pS6 fluorescence intensity, expressed as the injected/uninjected hemisphere ratio. Data are mean ± SEM; control, $n = 7$; *Pten*-deleted animals, $n = 8$; 3 sections per animal; unpaired t test.

(E) Co-localization of pS6 with TurboRFP or BDA labeling in the injected cortex. Arrowheads indicate double-positive neurons.

CellPress
OPEN ACCESS

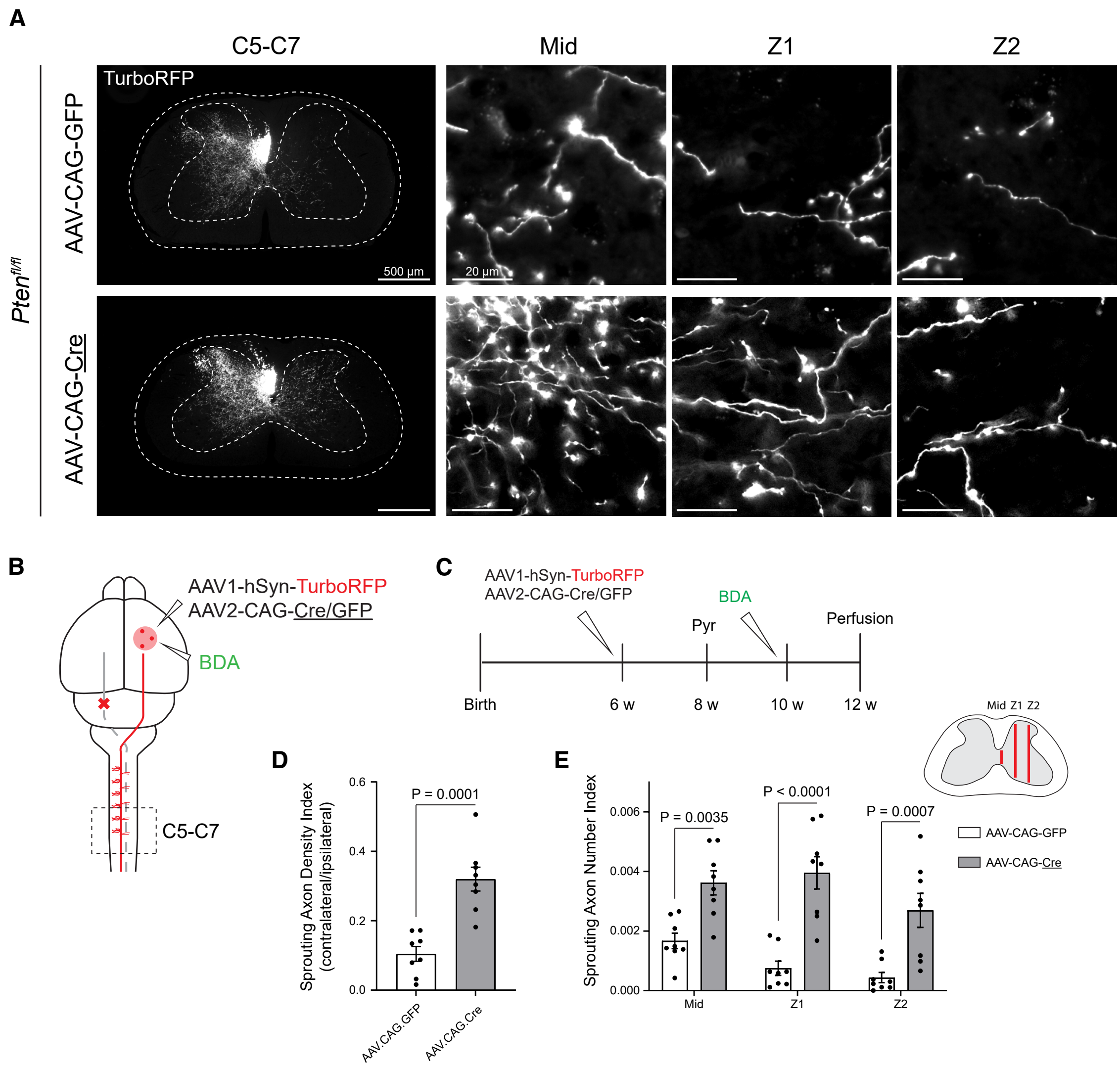

Figure 4. TurboRFP and BDA tracing of CST axons in *Pten*-deleted mice

(A) Representative C5–C7 spinal cord sections from $Pten^{fl/fl}$ mice showing TurboRFP-labeled CST terminals after cortical co-injection of AAV2-CAG-GFP or AAV2-CAG-Cre, with higher-magnification views of the denervated side.

(B) Schematic of cortical injection sites, unilateral pyramidotomy, and cervical CST sprouting analysis.

(C) Experimental timeline.

(D) Quantification of the sprouting axon density index, calculated as contralateral/ipsilateral axon density within the gray matter. Data are mean ± SEM; n = 8 animals per group, 5–6 sections per animal; unpaired t test.

(E) Quantification of the sprouting axon number index across Mid, Z1, and Z2 regions in the denervated gray matter.

Data are mean ± SEM; n = 8 animals per group, 5–6 sections per animal; two-way ANOVA with Šidák's multiple-comparisons test.

See also Figures S3–S6.

these findings support that TurboRFP can provide sufficiently strong native signal for CST tracing under appropriate conditions.

Advantages of TurboRFP over BDA for CST tracing and sprouting analysis

BDA injections performed in the same animals labeled substantially fewer cortical neurons and subsequently fewer CST axons (Figures 2G–2J). Although amplification-based BDA detection can improve sensitivity, it adds processing steps and staining-dependent variability. Consistent with this, the BDA labeling efficiency can exhibit substantial variability, even within the same laboratory over time.[23,26]

BDA tracing also often requires a second cortical injection shortly before tissue collection in longer sprouting paradigms, which may reduce labeling efficiency when prior cortical injections have already been performed. In contrast, TurboRFP can be co-injected with genetic manipulation vectors and provides sustained labeling throughout the experiment. With native fluorescence, TurboRFP labeling captured a large number of CST

axons, including thin or newly sprouting collateral fibers. This represents a major advantage for studies aiming to quantify axonal growth and response after injury, as these fine processes are often the most informative readout of plasticity. In this context, the key issue is not simply whether AAV-based tracers can label CST axons, but whether there is a practical and reproducible approach for reliably detecting sparse and fine sprouting CST axons within the spinal cord after injury.

Prior studies comparing AAV-based tracers and BDA have primarily focused on the cortical transduction efficiency and anatomical projection mapping within the brain.[39,40] Although these studies have demonstrated broad neuronal labeling with AAV-based approaches, they did not address the practical challenge of reliably detecting sparse CST collateral and sprouting axons within the spinal cord after injury. Similarly, while TurboRFP has previously been used to label CST axons,[34] that study did not establish or evaluate a simplified workflow for quantitative analysis of CST sprouting. By combining amplification-free TurboRFP labeling with a streamlined quantitative analysis pipeline, the current workflow enables sensitive detection and reproducible quantification of CST sprouting after injury.

Efficient viral delivery and compatibility with genetic manipulation

The workflow is also compatible with genetic manipulation. TurboRFP produced robust CST labeling across reduced injection volumes, supporting co-injection with Cre- or GFP-expressing AAVs. This avoids additional BDA surgery and helps align the labeled and genetically manipulated neuronal populations, which is important for interpreting circuit remodeling. In *Pten*-deleted mice, this signal-to-noise ratio enabled detection of increased CST sprouting after pyramidotomy.

Standardized and reproducible quantification

Coupling TurboRFP tracing with a semi-automated FeatureJ pipeline enabled standardized quantification of cervical CST sprouting (Figure S6). Hessian-based filtering enhanced axon-like structures, and consistent application of empirically optimized parameters reduced user-dependent variability.[27] Datasets with substantially different labeling patterns, such as sparse long-distance regeneration studies, may require modest adjustment of filtering or thresholding parameters.

Conclusion and outlook

In summary, this study establishes AAV1-hSyn-TurboRFP as a practical platform for CST sprouting analysis after unilateral pyramidotomy. Its main contribution is a simplified workflow that combines native fluorescence, compatibility with genetic manipulation, and standardized quantification to support reproducible assessment of corticospinal remodeling after injury. An expanding list of axonal tracers is emerging, such as codon-optimized membrane-embedded tracers (COMETs) built on existing fluorescent proteins,[41] which will further accelerate axon tracing and neural repair studies.

Limitations of the study

Although effective for detecting collateral sprouting after unilateral pyramidotomy, this workflow has not yet been fully validated for true CST regeneration after SCI. In such paradigms, pre-labeling before injury could complicate interpretation because degenerating distal axons may persist for weeks and resemble regenerating fibers.[42] Adapting AAV-TurboRFP to regeneration studies may, therefore, require optimization of viral delivery timing and post-injury survival intervals.

We also did not assess TurboRFP performance in very long-term experiments, and variability across experimental paradigms cannot be excluded. Native fluorescence was robust in fixed tissue but appeared less reliable in unfixed *ex vivo* preparations in our hands (not shown).

Finally, co-injection of different AAV serotypes may lead to incomplete overlap between the manipulated and labeled CSNs because serotypes differ in tropism and axonal transport.[9,16,43] Using the same serotype for reporter and functional constructs, when compatible with the experimental design, may improve co-transduction precision.

RESOURCE AVAILABILITY

Lead contact

Requests for further information, resources, and reagents should be directed to and will be fulfilled by the lead contact, Binhai Zheng (bizheng@health.ucsd.edu).

Materials availability

This study did not generate new unique reagents. All commercially available reagents and viral vectors used in this study are listed in the key resources table.

Data and code availability

- All data reported in this paper will be shared by the lead contact upon request.
- This paper does not report original code.
- Any additional information required to reanalyze the data reported in this paper is available from the lead contact upon request.

ACKNOWLEDGMENTS

The authors thank Geneva Le, Julieann Nodora, and Vincent Le for technical assistance. This work was supported by grants from the Wings for Life Spinal Cord Research Foundation (WFL-US-09/23 to M.H.-L. and WFL-US-27/24 to B.Z.), the National Institutes of Health (R01NS139527 and R01NS093055 to B.Z.) and the Craig H. Neilsen Foundation (733544 to B.Z.). C.J.L., E.W., C.L.C.-M., and C.K.A. have received support from the UC San Diego Genetics Training Program (T32GM145427 and T32GM008666). Some microscopy imaging during this research was performed at the UC San Diego School of Medicine Microscopy Core (P30NS047101, S10OD030505, and S10OD036455).

AUTHOR CONTRIBUTIONS

Conceptualization, M.H.-L. and B.Z.; methodology, M.H.-L., J.M.S., and B.Z.; investigation, M.H.-L., C.J.L., E.W., F.C., J.M.S., C.L.C.-M., H.J.K., and C.K.A.; formal analysis, M.H.-L.; visualization, M.H.-L.; resources, B.Z.; writing – original draft, M.H.-L. and B.Z.; writing – review & editing, all authors; supervision, B.Z.; funding acquisition, M.H.-L. and B.Z.

DECLARATION OF INTERESTS

The authors declare no competing interests.

DECLARATION OF GENERATIVE AI AND AI-ASSISTED TECHNOLOGIES IN THE WRITING PROCESS

During the preparation of this work, the authors used ChatGPT-5 to assist with language editing and formatting of manuscript and response materials. After

using this tool, the authors reviewed and edited the content as needed and take full responsibility for the content of the publication.

STAR★METHODS

Detailed methods are provided in the online version of this paper and include the following:

- KEY RESOURCES TABLE
- EXPERIMENTAL MODEL AND STUDY PARTICIPANT DETAILS
  - Animals
- METHOD DETAILS
  - Virus
  - AAV injections
  - Unilateral pyramidotomy
  - Biotinylated dextran amine (BDA) tracing
  - Tissue processing
  - Immunohistochemical staining
  - BDA staining
- QUANTIFICATION AND STATISTICAL ANALYSIS
  - Sprouting axon density index
  - Sprouting Axon Number Index
  - Total CST axon count (medulla)
  - pS6 intensity index (brain)
  - Statistics

SUPPLEMENTAL INFORMATION

Supplemental information can be found online at https://doi.org/10.1016/j.crmeth.2026.101540.

Received: March 31, 2026
Revised: May 6, 2026
Accepted: July 2, 2026
Published: July 31, 2026

REFERENCES

1. Martin, J.H. (2016). Harnessing neural activity to promote repair of the damaged corticospinal system after spinal cord injury. Neural Regen. Res. *11*, 1389–1391.
2. Steward, O., and Willenberg, R. (2017). Rodent spinal cord injury models for studies of axon regeneration. Exp. Neurol. *287*, 374–383. https://doi.org/10.1016/j.expneurol.2016.06.029.
3. Schwab, M.E., and Brösamle, C. (1997). Regeneration of lesioned corticospinal tract fibers in the adult rat spinal cord under experimental conditions. Spinal Cord *35*, 469–473. https://doi.org/10.1038/sj.sc.3100457.
4. Zheng, B., and Tuszynski, M.H. (2023). Regulation of axonal regeneration after mammalian spinal cord injury. Nat. Rev. Mol. Cell Biol. *24*, 396–413. https://doi.org/10.1038/s41580-022-00562-y.
5. Reiner, A., Veenman, C.L., Medina, L., Jiao, Y., Del Mar, N., and Honig, M.G. (2000). Pathway tracing using biotinylated dextran amines. J. Neurosci. Methods *103*, 23–37. https://doi.org/10.1016/S0165-0270(00)00293-4.
6. Tuszynski, M.H., and Steward, O. (2012). Concepts and Methods for the Study of Axonal Regeneration in the CNS. Neuron *74*, 777–791. https://doi.org/10.1016/j.neuron.2012.05.006.
7. Liu, K., Lu, Y., Lee, J.K., Samara, R., Willenberg, R., Sears-Kraxberger, I., Tedeschi, A., Park, K.K., Jin, D., Cai, B., et al. (2010). PTEN deletion enhances the regenerative ability of adult corticospinal neurons. Nat. Neurosci. *13*, 1075–1081. https://doi.org/10.1038/nn.2603.
8. Rosenzweig, E.S., Courtine, G., Jindrich, D.L., Brock, J.H., Ferguson, A.R., Strand, S.C., Nout, Y.S., Roy, R.R., Miller, D.M., Beattie, M.S., et al. (2010). Extensive spontaneous plasticity of corticospinal projections after primate spinal cord injury. Nat. Neurosci. *13*, 1505–1510. https://doi.org/10.1038/nn.2691.
9. Chan, K.Y., Jang, M.J., Yoo, B.B., Greenbaum, A., Ravi, N., Wu, W.-L., Sánchez-Guardado, L., Lois, C., Mazmanian, S.K., Deverman, B.E., and Gradinaru, V. (2017). Engineered AAVs for efficient noninvasive gene delivery to the central and peripheral nervous systems. Nat. Neurosci. *20*, 1172–1179. https://doi.org/10.1038/nn.4593.
10. Zingg, B., Chou, X.L., Zhang, Z.G., Mesik, L., Liang, F., Tao, H.W., and Zhang, L.I. (2017). AAV-Mediated Anterograde Transsynaptic Tagging: Mapping Corticocollicular Input-Defined Neural Pathways for Defense Behaviors. Neuron *93*, 33–47. https://doi.org/10.1016/j.neuron.2016.11.045.
11. Blackmore, M.G., Wang, Z., Lerch, J.K., Motti, D., Zhang, Y.P., Shields, C.B., Lee, J.K., Goldberg, J.L., Lemmon, V.P., and Bixby, J.L. (2012). Krüppel-like Factor 7 engineered for transcriptional activation promotes axon regeneration in the adult corticospinal tract. Proc. Natl. Acad. Sci. USA *109*, 7517–7522. https://doi.org/10.1073/pnas.1120684109.
12. Liu, Y., Wang, X., Li, W., Zhang, Q., Li, Y., Zhang, Z., Zhu, J., Chen, B., Williams, P.R., Zhang, Y., et al. (2017). A Sensitized IGF1 Treatment Restores Corticospinal Axon-Dependent Functions. Neuron *95*, 817–833.e4. https://doi.org/10.1016/j.neuron.2017.07.037.
13. Hutson, T.H., Verhaagen, J., Yáñez-Muñoz, R.J., and Moon, L.D.F. (2012). Corticospinal tract transduction: A comparison of seven adeno-associated viral vector serotypes and a non-integrating lentiviral vector. Gene Ther. *19*, 49–60. https://doi.org/10.1038/gt.2011.71.
14. Nieuwenhuis, B., Haenzi, B., Hilton, S., Carnicer-Lombarte, A., Hobo, B., Verhaagen, J., and Fawcett, J.W. (2021). Optimization of adeno-associated viral vector-mediated transduction of the corticospinal tract: comparison of four promoters. Gene Ther. *28*, 56–74. https://doi.org/10.1038/s41434-020-0169-1.
15. Xu, X., Holmes, T.C., Luo, M.-H., Beier, K.T., Horwitz, G.D., Zhao, F., Zeng, W., Hui, M., Semler, B.L., and Sandri-Goldin, R.M. (2020). Viral Vectors for Neural Circuit Mapping and Recent Advances in Trans-synaptic Anterograde Tracers. Neuron *107*, 1029–1047. https://doi.org/10.1016/j.neuron.2020.07.010.
16. Aschauer, D.F., Kreuz, S., and Rumpel, S. (2013). Analysis of Transduction Efficiency, Tropism and Axonal Transport of AAV Serotypes 1, 2, 5, 6, 8 and 9 in the Mouse Brain. PLoS One *8*, e76310.
17. Castle, M.J., Perlson, E., Holzbaur, E.L., and Wolfe, J.H. (2014). Long-distance Axonal Transport of AAV9 Is Driven by Dynein and Kinesin-2 and Is Trafficked in a Highly Motile Rab7-positive Compartment. Mol. Ther. *22*, 554–566. https://doi.org/10.1038/mt.2013.237.
18. Salegio, E.A., Samaranch, L., Kells, A.P., Mittermeyer, G., San Sebastian, W., Zhou, S., Beyer, J., Forsayeth, J., and Bankiewicz, K.S. (2013). Axonal transport of adeno-associated viral vectors is serotype-dependent. Gene Ther. *20*, 348–352. https://doi.org/10.1038/gt.2012.27.
19. Issa, S.S., Shaimardanova, A.A., Solovyeva, V.V., and Rizvanov, A.A. (2023). Various AAV Serotypes and Their Applications in Gene Therapy: An Overview. Cells *12*, 785. https://doi.org/10.3390/cells12050785.
20. Kügler, S., Kilic, E., and Bähr, M. (2003). Human synapsin 1 gene promoter confers highly neuron-specific long-term transgene expression from an adenoviral vector in the adult rat brain depending on the transduced area. Gene Ther. *10*, 337–347. https://doi.org/10.1038/sj.gt.3301905.
21. Merzlyak, E.M., Goedhart, J., Shcherbo, D., Bulina, M.E., Shcheglov, A.S., Fradkov, A.F., Gaintzeva, A., Lukyanov, K.A., Lukyanov, S., Gadella, T.W.J., and Chudakov, D.M. (2007). Bright monomeric red fluorescent protein with an extended fluorescence lifetime. Nat. Methods *4*, 555–557. https://doi.org/10.1038/nmeth1062.
22. Ueno, M., Nakamura, Y., Li, J., Gu, Z., Niehaus, J., Maezawa, M., Crone, S.A., Goulding, M., Baccei, M.L., and Yoshida, Y. (2018). Corticospinal Circuits from the Sensory and Motor Cortices Differentially Regulate Skilled Movements through Distinct Spinal Interneurons. Cell Rep. *23*, 1286–1300.e7. https://doi.org/10.1016/j.celrep.2018.03.137.

23. Geoffroy, C.G., Lorenzana, A.O., Kwan, J.P., Lin, K., Ghassemi, O., Ma, A., Xu, N., Creger, D., Liu, K., He, Z., and Zheng, B. (2015). Effects of PTEN and Nogo Codeletion on Corticospinal Axon Sprouting and Regeneration in Mice. J. Neurosci. *35*, 6413.LP–6428. https://doi.org/10.1523/JNEUROSCI.4013-14.2015.

24. Park, K.K., Liu, K., Hu, Y., Smith, P.D., Wang, C., Cai, B., Xu, B., Connolly, L., Kramvis, I., Sahin, M., and He, Z. (2008). Promoting Axon Regeneration in the Adult CNS by Modulation of the PTEN/mTOR Pathway. Science *322*, 963–966. https://doi.org/10.1126/science.1161566.

25. Meves, J.M., and Zheng, B. (2014). Extrinsic inhibitors in axon sprouting and functional recovery after spinal cord injury. Neural Regen. Res. *9*, 460–461.

26. Saikia, J.M., Chavez-Martinez, C.L., Kim, N.D., Allibhoy, S., Kim, H.J., Simonyan, L., Smadi, S., Tsai, K.M., Romaus-Sanjurjo, D., Jin, Y., and Zheng, B. (2022). A Critical Role for DLK and LZK in Axonal Repair in the Mammalian Spinal Cord. J. Neurosci. *42*, 3716–3732. https://doi.org/10.1523/JNEUROSCI.2495-21.2022.

27. Grider, M.H., Chen, Q., and Shine, H.D. (2006). Semi-automated quantification of axonal densities in labeled CNS tissue. J. Neurosci. Methods *155*, 172–179. https://doi.org/10.1016/j.jneumeth.2005.12.021.

28. Fink, K.L., Strittmatter, S.M., and Cafferty, W.B.J. (2015). Comprehensive corticospinal labeling with mu-crystallin transgene reveals axon regeneration after spinal cord trauma in ngr1−/− mice. J. Neurosci. *35*, 15403–15418. https://doi.org/10.1523/JNEUROSCI.3165-15.2015.

29. Balena, T., Lillis, K., Rahmati, N., Bahari, F., Dzhala, V., Berdichevsky, E., and Staley, K. (2023). A Dynamic Balance between Neuronal Death and Clearance in an in Vitro Model of Acute Brain Injury. J. Neurosci. *43*, 6084–6107. https://doi.org/10.1523/JNEUROSCI.0436-23.2023.

30. Dingle, Y.T.L., Liaudanskaya, V., Finnegan, L.T., Berlind, K.C., Mizzoni, C., Georgakoudi, I., Nieland, T.J.F., and Kaplan, D.L. (2020). Functional Characterization of Three-Dimensional Cortical Cultures for In Vitro Modeling of Brain Networks. iScience *23*, 101434. https://doi.org/10.1016/j.isci.2020.101434.

31. Leiwe, M.N., Fujimoto, S., Baba, T., Moriyasu, D., Saha, B., Sakaguchi, R., Inagaki, S., and Imai, T. (2024). Automated neuronal reconstruction with super-multicolour Tetbow labelling and threshold-based clustering of colour hues. Nat. Commun. *15*, 5279. https://doi.org/10.1038/s41467-024-49455-y.

32. Fujita, H., Kodama, T., and Du Lac, S. (2020). Modular output circuits of the fastigial nucleus for diverse motor and nonmotor functions of the cerebellar vermis. eLife *9*, e58613–e58691. https://doi.org/10.7554/eLife.58613.

33. Georgiou, L., Echeverría, A., Georgiou, A., and Kuhn, B. (2022). Ca 2+ Activity Maps of Astrocytes Tagged by Axoastrocytic AAV Transfer. Sci Adv *8*, eabe5371. https://doi.org/10.1126/sciadv.abe5371.

34. Itoh, Y., Sahni, V., Shnider, S.J., McKee, H., and Macklis, J.D. (2023). Interaxonal molecular crosstalk via Lumican proteoglycan sculpts murine cervical corticospinal innervation by distinct subpopulations. Cell Rep. *42*, 112182. https://doi.org/10.1016/j.celrep.2023.112182.

35. Mesías, R.E., Zaki, Y., Guevara, C.A., Friedman, L.G., Hussein, A., Therrien, K., Magee, A.R., Tzavaras, N., Del Valle, P., Baxter, M.G., et al. (2023). Development and cadherin-mediated control of prefrontal cortico-striatal projections in mice. iScience *26*, 108002. https://doi.org/10.1016/j.isci.2023.108002.

36. Sekine, Y., Kannan, R., Wang, X., and Strittmatter, S.M. (2022). Rabphilin3A reduces integrin-dependent growth cone signaling to restrict axon regeneration after trauma. Exp. Neurol. *353*, 114070. https://doi.org/10.1016/j.expneurol.2022.114070.

37. Tezuka, Y., Hagihara, K.M., Ohki, K., Hirano, T., and Tagawa, Y. (2022). Developmental stage-specific spontaneous activity contributes to callosal axon projections. eLife *11*, 72435. https://doi.org/10.7554/eLife.

38. Squair, J.W., Milano, M., de Coucy, A., Gautier, M., Skinnider, M.A., James, N.D., Cho, N., Lasne, A., Kathe, C., Hutson, T.H., et al. (2023). Recovery of walking after paralysis by regenerating characterized neurons to their natural target region. Science *381*, 1338–1345. https://doi.org/10.1126/science.adi6412.

39. Oh, S.W., Harris, J.A., Ng, L., Winslow, B., Cain, N., Mihalas, S., Wang, Q., Lau, C., Kuan, L., Henry, A.M., et al. (2014). A mesoscale connectome of the mouse brain. Nature *508*, 207–214. https://doi.org/10.1038/nature13186.

40. Wang, Q., Henry, A.M., Harris, J.A., Oh, S.W., Joines, K.M., Nyhus, J., Hirokawa, K.E., Dee, N., Mortrud, M., Parry, S., et al. (2014). Systematic comparison of adeno-associated virus and biotinylated dextran amine reveals equivalent sensitivity between tracers and novel projection targets in the mouse brain. J. Comp. Neurol. *522*, 1989–2012. https://doi.org/10.1002/cne.23567.

41. Dulin, J.N., Ye, L., Grider, T., Rizzo, A., Rosenzweig, E.S., Weber, J., Ramakrishnan, C., Hsueh, B., Deisseroth, K., Tuszynski, M.H., et al. (2026). Optimized genetic tracers for viral mediated neuronal projection mapping. Cell. Mol. Biol. Lett. *31*, 53. https://doi.org/10.1186/s11658-026-00894-z.

42. Schading, S., David, G., Max Emmenegger, T., Achim, C., Thompson, A., Weiskopf, N., Curt, A., and Freund, P. (2023). Dynamics of progressive degeneration of major spinal pathways following spinal cord injury: A longitudinal study. Neuroimage, Clin. *37*, 103339. https://doi.org/10.1016/j.nicl.2023.103339.

43. Tervo, D.G.R., Hwang, B.-Y., Viswanathan, S., Gaj, T., Lavzin, M., Ritola, K.D., Lindo, S., Michael, S., Kuleshova, E., Ojala, D., et al. (2016). A Designer AAV Variant Permits Efficient Retrograde Access to Projection Neurons. Neuron *92*, 372–382. https://doi.org/10.1016/j.neuron.2016.09.021.

44. Kim, H.J., Saikia, J.M., Monte, K.M.A., Ha, E., Romaus-Sanjurjo, D., Sanchez, J.J., Moore, A.X., Hernaiz-Llorens, M., Chavez-Martinez, C.L., Agba, C.K., et al. (2023). Deep scRNA sequencing reveals a broadly applicable Regeneration Classifier and implicates antioxidant response in corticospinal axon regeneration. Neuron *111*, 3953–3969.e5. https://doi.org/10.1016/j.neuron.2023.09.019.

45. Schneider, C.A., Rasband, W.S., and Eliceiri, K.W. (2012). NIH Image to ImageJ: 25 years of image analysis. Nat. Methods *9*, 671–675. https://doi.org/10.1038/nmeth.2089.

46. Meijering, E. (2015). FeatureJ: An ImageJ plugin suite for image feature extraction. https://imagescience.org/meijering/software/featurej/.

47. Gallent, E.A., and Steward, O. (2018). Neuronal PTEN deletion in adult cortical neurons triggers progressive growth of cell bodies, dendrites, and axons. Exp. Neurol. *303*, 12–28. https://doi.org/10.1016/j.expneurol.2018.01.005.

CellPress
OPEN ACCESS

STAR★METHODS

KEY RESOURCES TABLE

REAGENT or RESOURCE	SOURCE	IDENTIFIER
**Antibodies**		
Rabbit monoclonal anti-PKCγ	Cell Signaling	Cat# 59090; RRID:AB_2799557
Rabbit polyclonal anti-pS6	Cell Signaling	Cat# 2211; RRID:AB_331679
Rabbit polyclonal anti-RFP	Rockland	Cat# 600-401-379; RRID:AB_2209751
Goat anti-Rabbit Alexa Fluor 488	Thermo Fisher Scientific	Cat# A-11008; RRID:AB_143165
Goat anti-Rabbit Alexa Fluor 647	Thermo Fisher Scientific	Cat# A-21245; RRID:AB_2535813
**Bacterial and virus strains**		
AAV2.CAG-Cre-WPRE	Boston Children's Hospital	N/A
AAV2.CAG-GFP-WPRE	Boston Children's Hospital	N/A
AAV1.hSyn.TurboRFP.WPRE.RBG	Addgene	Cat# 105552-AAV1
**Chemicals, peptides, and recombinant proteins**		
Biotinylated Dextran, 10,000 MW	Thermo Fisher Scientific	Cat# D1956; RRID:AB_2307337
DAPI	Thermo Fisher Scientific	Cat# D1306
Fluoromount-G	Southern Biotechnology	Cat# 0100-01
Alexa Fluor 405 Streptavidin	Thermo Fisher Scientific	Cat# S32351
Alexa Fluor 488 Streptavidin	Thermo Fisher Scientific	Cat# S11223
**Experimental models: Organisms/strains**		
Mouse: *Pten*$^{fl/fl}$; B6.129S4-*Pten*^{tm1Hwu}/J	The Jackson Laboratory	Strain#: 006440
Mouse: C57BL/6NCrl	Charles River	Strain#: 027
**Software and algorithms**		
GraphPad Prism version 10.3.0	GraphPad software Inc.	https://www.graphpad.com/
ImageJ 2.9.0	NIH	https://github.com/imagej/imagej1
Zen Blue	Zeiss	N/A
FeatureJ	ImageScience	http://imagescience.org/meijering/software/featurej/
**Other**		
Nanoject III™	Drummond Scientific Company	Cat# 3-000-207
Nanoject Glass Capillaries	Drummond Scientific Company	Cat# 3-000-203-G/X
Feather™ Sterile MicroScalpels	Electron Microscopy Sciences	Cat# 72045-15
Tissue-Tek® O.C.T. Compound	Sakura	Cat# 4583
ImmEdge Hydrophobic Barrier (PAP) Pen	Vector Laboratories	Cat# H-4000

EXPERIMENTAL MODEL AND STUDY PARTICIPANT DETAILS

Animals

Wild-type mice were obtained from Charles River Laboratories. *Pten* conditional knockout (cKO) mice (*Pten*$^{fl/fl}$) on a C57BL/6 background were used. AAV-Cre was used to conditionally delete *Pten*. To verify the efficacy of *Pten* deletion, immunostaining for phospho-S6 (pS6), a readout of mTOR signaling activity, was performed in all experimental animals.

For sprouting assays, age-matched *Pten*$^{fl/fl}$ mice received injections of AAV2-CAG-Cre or AAV2-CAG-GFP into the right sensorimotor cortex to induce gene deletion or serve as controls, respectively. Both male and female mice were used at an approximately 1:1 ratio.

METHOD DETAILS

Virus

AAV2-CAG-Cre and AAV2-CAG-GFP were produced at the Boston Children's Hospital Viral Core. Viral titers were determined by qPCR and were 1×10^{13} IU/mL. pENN.AAV.hSyn.TurboRFP.WPRE.RBG was a gift from James M. Wilson (Addgene viral prep # 105552-AAV1; http://n2t.net/addgene:105552; RRID:Addgene_105552). Viral titer was $\geq 1 \times 10^{13}$ IU/mL.

AAV injections

Cortical co-injections of AAV-Cre or AAV-GFP with AAV-TurboRFP were performed in six-week-old mice using a programmable nanoliter injector (Nanoject III™, Drummond Scientific) coupled with 3.5″ Drummond glass capillaries (3-000-203-G/X). Capillaries were pulled using a laser-based puller to generate fine injection needles.

Mice were anesthetized by intraperitoneal (i.p.) injection of ketamine (5 mg/mL) and xylazine (0.5 mg/mL) in saline at a dose of 0.02 mL/g body weight. Animals were then shaved at the surgical site and secured in a stereotaxic frame. The surgical area was sterilized using 70% isopropyl alcohol wipes. An incision was made in the scalp to expose bregma. A cranial window was drilled relative to bregma to target three injection sites.

Glass capillary needles were loaded with up to 5 μL of viral mixture consisting of AAV2-CAG-Cre or AAV2-CAG-GFP mixed with AAV1-hSyn-TurboRFP at a 1:1 ratio, supplemented with 200 nL Fast Green FCF to visualize injection volume. Needles were mounted on the stereotaxic apparatus for delivery. Injection coordinates were adapted from prior studies targeting the forelimb motor cortex in the pyramidotomy model.[44] Coordinates relative to bregma were: (1) 1.2 mm lateral, 0.5 mm anterior; (2) 1.2 mm lateral, 0.5 mm posterior; and (3) 2.2 mm lateral, 0.0 mm anterior. For each site, the needle was lowered to a depth of 0.7 mm. After positioning, tissue was allowed to equilibrate for 2 min prior to injection. Virus was delivered at a rate of 1 nL/s for a total volume of 400 nL per injection site. The needle was left in place for an additional 4 min to allow diffusion before withdrawal.

After injection, the incision was closed using 7 mm Reflex wound clips. Mice were monitored postoperatively and returned to their home cages, which were maintained on a circulating warm water heating pad. For analgesia, mice received subcutaneous injections of 0.5 mL saline and buprenorphine (0.05–0.1 mg/kg) for pain management. Wound clips were removed 14 days after surgery.

Unilateral pyramidotomy

Pyramidotomy was performed two weeks after AAV injection following established protocols.[26] Mice were anesthetized by intraperitoneal (i.p.) injection of ketamine/xylazine and placed in a supine position under a dissection microscope. A ~2 cm midline incision was made to expose the esophagus and adjacent neck musculature. Blunt dissection was used to access the ventral medulla and visualize the medullary pyramids while avoiding damage to blood vessels. A 15° microscalpel (Feather; Electron Microscopy Sciences, 72045-15) was used to make a ~0.7 mm deep incision to transect the left medullary pyramid rostral to the decussation. The incision depth was controlled to restrict the lesion to the targeted pyramid. The wound was closed using tissue adhesive (Vetbond, 3M), and mice were allowed to recover with heat support and postoperative pain management.

Biotinylated dextran amine (BDA) tracing

Two weeks after pyramidotomy, 400 nL of the anterograde tracer BDA (10%, 10,000 MW, in sterile PBS) were injected into three different sites of the forelimb motor cortex using the same stereotaxic coordinates described above. Wound closure, recovery, and postoperative care were performed as described for AAV injections.

Tissue processing

Experimental animals were euthanized with a lethal dose of pentobarbital sodium (Fatal Plus) and transcardially perfused with ice-cold 4% paraformaldehyde (PFA) in sterile PBS for 10 min. Brain and spinal cord tissues were dissected and post-fixed in 4% PFA overnight at 4°C. Tissues were then transferred to 30% sucrose in PBS and incubated at 4°C for 48 h for cryoprotection, followed by embedding in Tissue-Tek OCT compound (Sakura, 4583) and freezing on dry ice.

For sprouting assays, brain, medulla, and C5–C7 spinal cord segments were collected and sectioned transversely at 20 μm thickness using a cryostat.

Immunohistochemical staining

Cervical spinal cord sections were immunostained for PKCγ (1:200; rabbit monoclonal anti-PKCγ, Cell Signaling, 59090) to assess completeness of the pyramidotomy lesion. Animals exhibiting bilateral PKCγ signal in the CST were excluded from analysis. Coronal brain sections were immunostained for phospho-S6 (pS6; 1:200; rabbit polyclonal anti-pS6, Cell Signaling, 2211). Coronal brain sections, as well as transverse sections of the medulla and cervical spinal cord, were immunostained for RFP (1:200; rabbit polyclonal anti-RFP, Rockland, 600-401-379).

Sections were dried for 15 min at 40°C and rinsed in PBS containing Triton X-100 (PBS-Tx). Samples were blocked in 5% normal goat serum (NGS) in PBS-Tx for 1 h at room temperature with gentle agitation. Primary antibodies were diluted in 2% NGS in PBS-Tx

and incubated overnight at 4°C. Sections were then incubated with goat anti-rabbit secondary antibodies conjugated to Alexa Fluor 647 or Alexa Fluor 488 (1:200), along with DAPI (1:2000), for 1 h at room temperature. After three washes in PBS-Tx, sections were mounted using Fluoromount-G and coverslipped.

Transverse sections of cervical spinal cord and medulla were collected free-floating in PBS and mounted onto Superfrost Plus slides (Thermo Fisher Scientific). Sections were dried at 40°C for 15 min, rinsed once in PBS containing 0.4% Triton X-100 (PBS-Tx), and incubated with DAPI (1:2000 in PBS-Tx) for 10 min. Slides were then washed three times in PBS-Tx (10 min each) and mounted using Fluoromount-G and coverslipped.

BDA staining

BDA detection was performed using fluorescently conjugated streptavidin without ABC or TSA amplification. Brain, medulla, and cervical spinal cord sections were processed free-floating for BDA detection. Sections were permeabilized with two 30-min washes in 0.4% PBS-Tx. Sections were then incubated with a fluorescently labeled streptavidin (1:200), either Alexa Fluor 488 or 405, overnight at 4°C. The following day, sections were washed three times in 0.4% PBS-Tx and mounted using Fluoromount-G and coverslipped.

QUANTIFICATION AND STATISTICAL ANALYSIS

Sprouting axon density index

Transverse sections were mounted onto Superfrost Plus slides (Thermo Fisher Scientific) and counterstained with DAPI prior to imaging. For each animal, at least five transverse sections from C5–C7 spinal cord levels were imaged at three focal planes using an upright microscope (Zeiss Axio Imager M1) with a 10× objective.

Images were analyzed using FIJI.[45] Labeled CST axons were detected using the FeatureJ plugin (Erik Meijering; https://imagescience.org/meijering/software/featurej/),[8,27] which enhances detection of tubular structures such as axons while reducing background signal.[46] Axons were identified using the "smallest eigenvalue of the Hessian tensor" with a smoothing scale of 2, a parameter empirically optimized using representative sections by comparison with manual axon identification. Once optimized, the same parameters were applied consistently across all sections and experimental groups within each dataset to reduce user-dependent variability and improve reproducibility. Processed images were subsequently thresholded and binarized for quantitative analysis. Minor adjustment of thresholding parameters may be required for datasets with substantially different signal-to-noise ratios or labeling densities.

The area occupied by binarized axons was quantified separately for the contralateral (denervated) and ipsilateral (intact) sides. Sprouting density was expressed as a ratio of contralateral to ipsilateral signal. Values from individual sections were averaged to obtain a per-animal measurement (Figure S6).

$$\textit{Sprouting Axon Density Index} = \frac{\textit{Contralateral axon density}}{\textit{Ipsilateral axon density}}$$

Sprouting Axon Number Index

Using the same image processing pipeline described for sprouting density, three sampling boxes (5 μm in width), spaced 245 μm apart, were positioned from the central canal (Mid) to the lateral gray matter (Z2). Axons crossing each box were quantified using the FIJI particle analyzer tool. Counts from the three regions (Mid, Z1, Z2) were averaged across at least five sections per animal. Quantification was adapted from K. Liu et al., 2010[7] to accommodate the higher density of labeled axons in AAV-traced spinal cords. Axon counts were normalized to the total number of labeled CST axons measured at the medulla level. Sprouting was expressed as a normalized axon number index (Figure S6).

$$\textit{Sprouting Axon Number Index} = \frac{\textit{Number of axons crossing Mid}, Z1, Z2}{\textit{total CST axon count at medulla}}$$

Total CST axon count (medulla)

Total CST axon counts at the medulla level were measured for normalization of sprouting indices. Medulla sections were mounted onto Superfrost Plus slides (Thermo Fisher Scientific) and counterstained with DAPI. For each animal, at least three sections were imaged using a 100× oil-immersion objective and analyzed in FIJI.

Axons were quantified using the Find Maxima plugin, which detects local intensity maxima based on a defined prominence threshold. Prominence values were empirically optimized by comparison with manual axon counts, and the selected threshold was applied uniformly across all experimental samples.[7]

pS6 intensity index (brain)

Coronal brain sections encompassing the cortical injection sites were immunostained for pS6 as a readout of *Pten* deletion. For each animal, at least three sections were analyzed. Signal intensity was quantified in layer V of each hemisphere, identified based on GFP expression or cytoarchitectural features.[47]

Regions of interest (ROIs; 300 × 300 μm) were placed in each hemisphere at equivalent distances from the midline. After background subtraction, pS6 signal was thresholded and segmented to quantify total signal area and mean intensity. These values were multiplied to obtain an integrated intensity measure. The pS6 intensity index was calculated as the ratio of signal in the injected hemisphere to that in the uninjected hemisphere.[26]

Statistics

Statistical analyses were performed using GraphPad Prism. Data are presented as mean ± SEM unless otherwise indicated. The number of animals and sections analyzed for each experiment is indicated in the corresponding figure legends. For within-animal comparisons between TurboRFP and BDA labeling, paired two-tailed Student's t-tests were used. For comparisons between two independent groups, unpaired two-tailed Student's t-tests were used. For comparisons involving multiple regions or factors, two-way ANOVA followed by Šidák's multiple comparisons test was used. Statistical significance was defined as $p < 0.05$. No statistical methods were used to predetermine sample size. Animals with incomplete pyramidotomy lesions, as assessed by residual PKCγ immunoreactivity in the injured CST, were excluded from analysis.

Cell Reports Methods, Volume *6*

Supplemental information

AAV-delivered TurboRFP enables streamlined tracing and analysis of corticospinal tract sprouting

Marc Hernaiz-Llorens, Camilo J. Londoño, Ellen Wang, Felicia Cai, Junmi M. Saikia, Carmine L. Chavez-Martinez, Hugo J. Kim, Chimuanya K. Agba, and Binhai Zheng

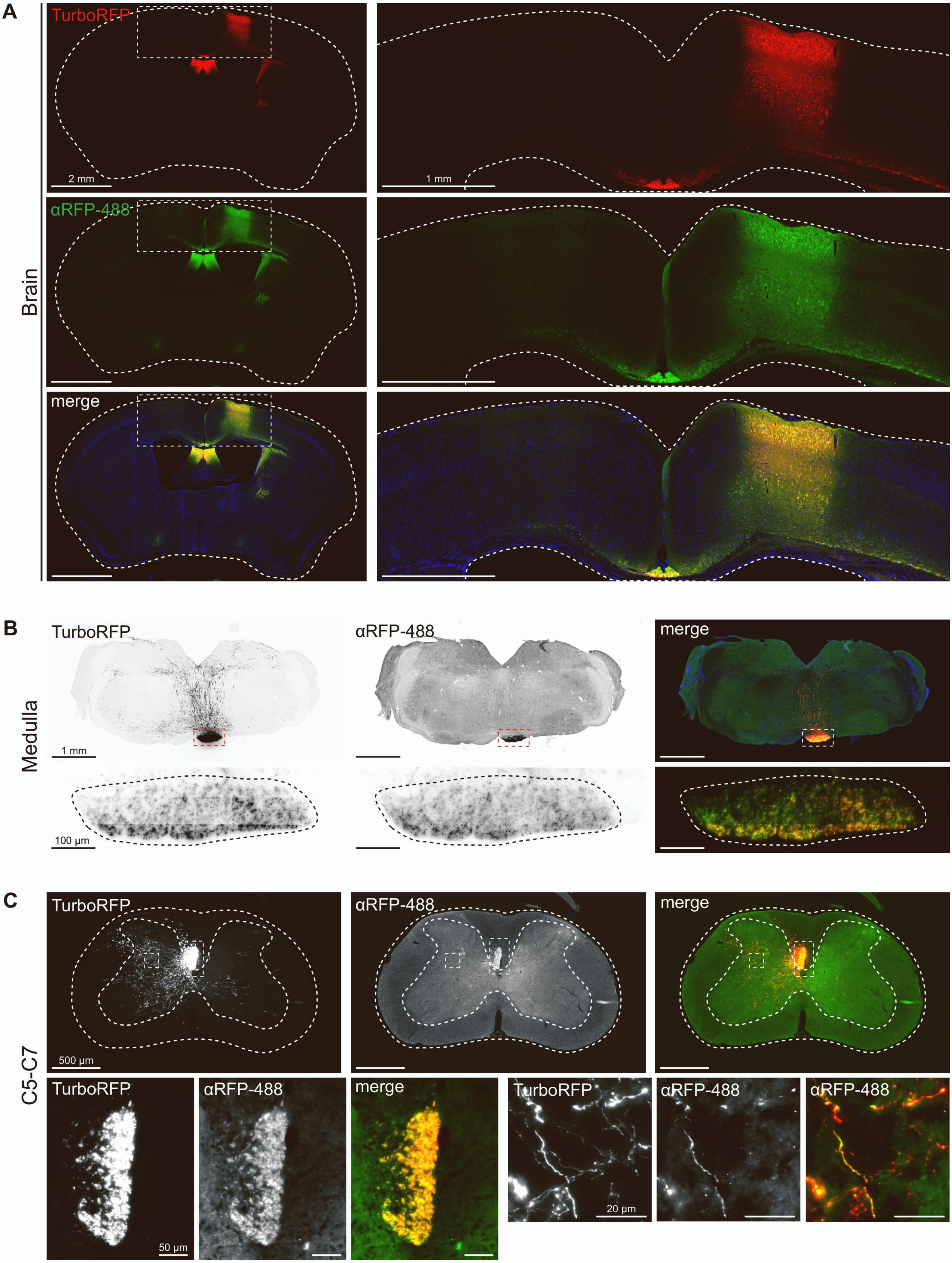

Figure S1. Immunodetection of TurboRFP tracing along the CST. Related to Figure 1, 2 and 4. (A) Coronal brain sections showing TurboRFP labeling (red) and corresponding αRFP-488 immunostaining (green). Merged images confirm largely overlapping signals. The same color scheme applies for merged images below. **(B)** Transversal medulla sections showing TurboRFP-labeled CST axons and overlapping αRFP-488 immunostaining signal. **(C)** Transversal cervical spinal cord sections (C5-C7) showing CST and collateral fibers labeled with TurboRFP. Immunolabeling with αRFP-488 showed clear signal within the main CST but did not improve visualization of fine CST collaterals or varicosities in the cervical spinal cord and introduced substantial background signal under our experimental conditions. Higher power insets show details of native TurboRFP and immunodetected TurboRFP signals in the main CST and collateral fibers.

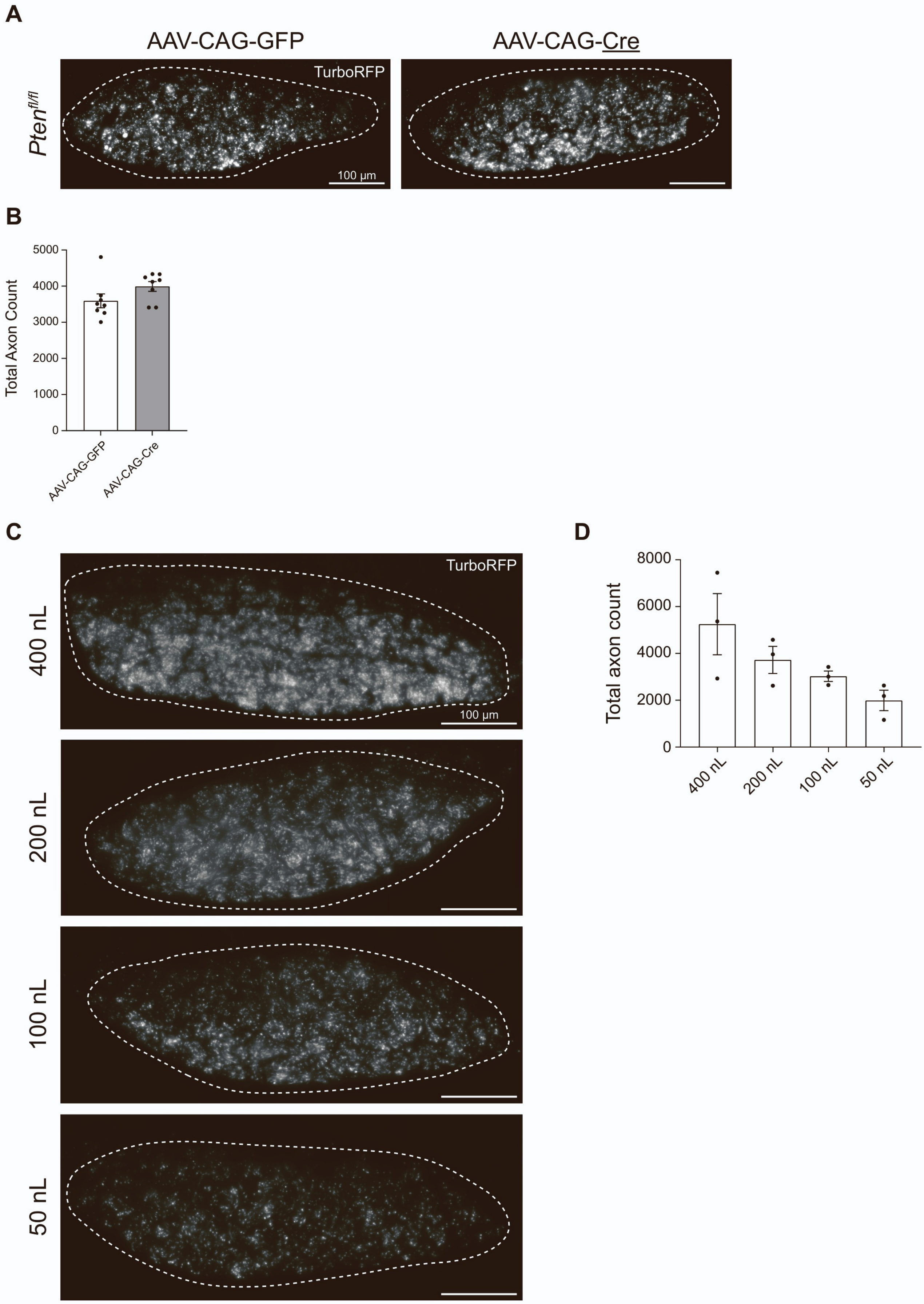

Figure S2. Effects of Pten deletion and AAV1-hSyn-TurboRFP injection volume on total CST axon counts in the medulla. Related to Figures 2, 3, and 4. (A) Representative transverse sections of TurboRFP traced axons in the CST at the medullary pyramid level from Ptenfl/fl mice injected with either AAV2-CAG-GFP (control) or AAV2-CAG-Cre, showing comparable TurboRFP-labeled axons. **(B)** Quantification of total TurboRFP-positive axons at the medullary level showing no significant difference between AAV-CAG-GFP and AAV-CAG-Cre injected groups. Results are presented as mean ± SEM (n = 8 animals per experimental group, unpaired t-test). **(C)** Representative transverse sections of TurboRFP traced axons in the CST at the medullary pyramid level injected with different volumes of AAV1-hSyn-TurboRFP (400 nL, 200 nL, 100 nL or 50 nL per injection site) into the cortex of wild-type animals. **(D)** Quantification of total CST axon counts across injection volumes. Although no statistically significant differences were detected across the different injection volumes, there was a trend for a modest decline in labeling efficiency as injection volume reduced. Results are presented as mean ± SEM (n = 3 animals per experimental group, 3 medulla sections analyzed per animal, one-way ANOVA with post-hoc Tukey's multiple comparison test).

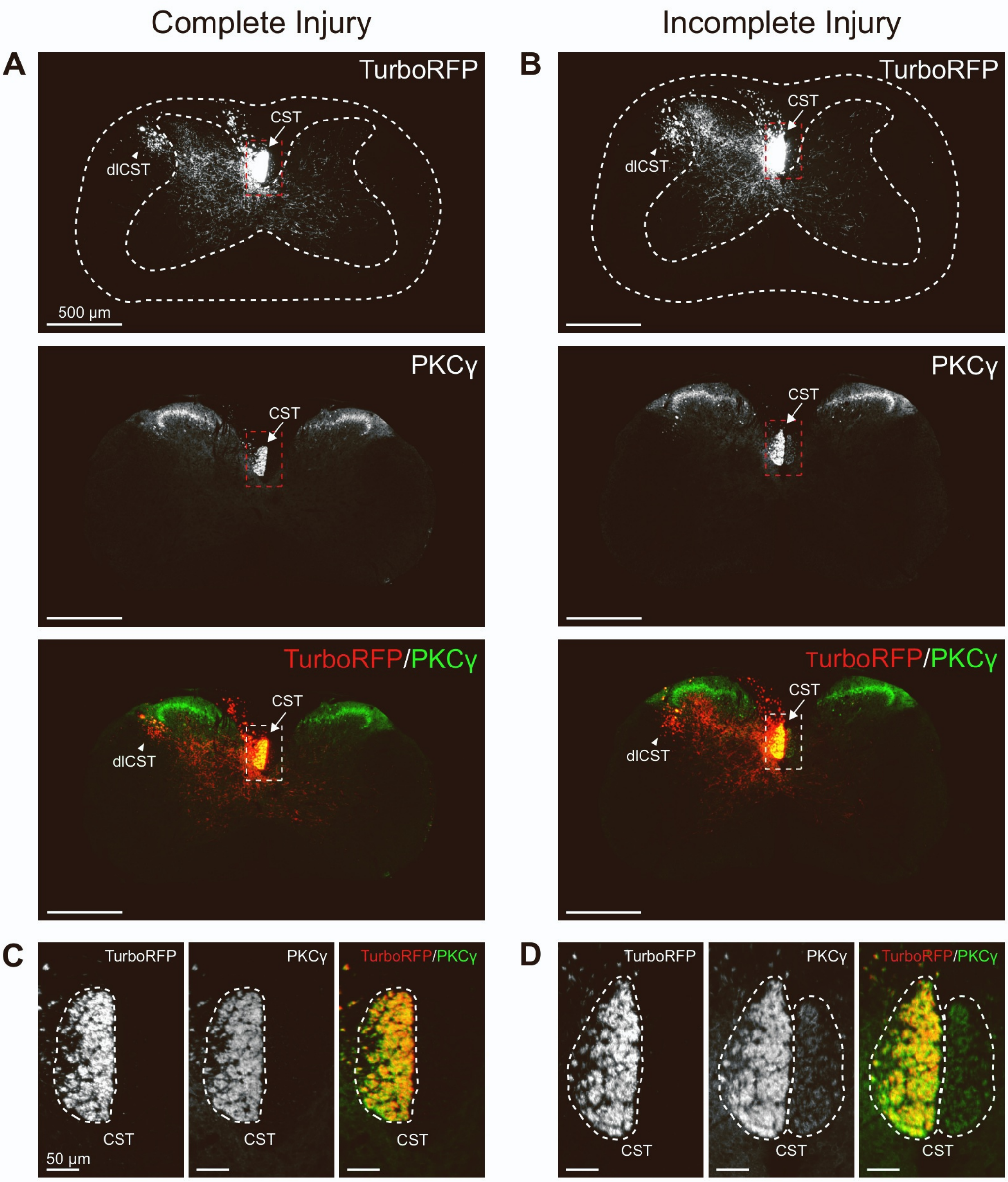

Figure S3. Verification of the pyramidotomy lesion completeness by PKCγ detection. Related to Figure 4. (A) Representative transverse sections of the cervical spinal cord (C5-C7) from an animal with a complete injury, showing TurboRFP labeling of the intact CST, collateral projections and the dorsolateral CST (dlCST) and loss of PKCγ immunoreactivity within the lesioned CST. **(B)** Example of an incomplete injury, showing residual PKCγ immunoreactivity within the partially injured CST. (C, D) Higher-magnification views of the CST region in complete **(C)** and incomplete **(D)** injuries, illustrating the absence or persistence of PKCγ signal, respectively.

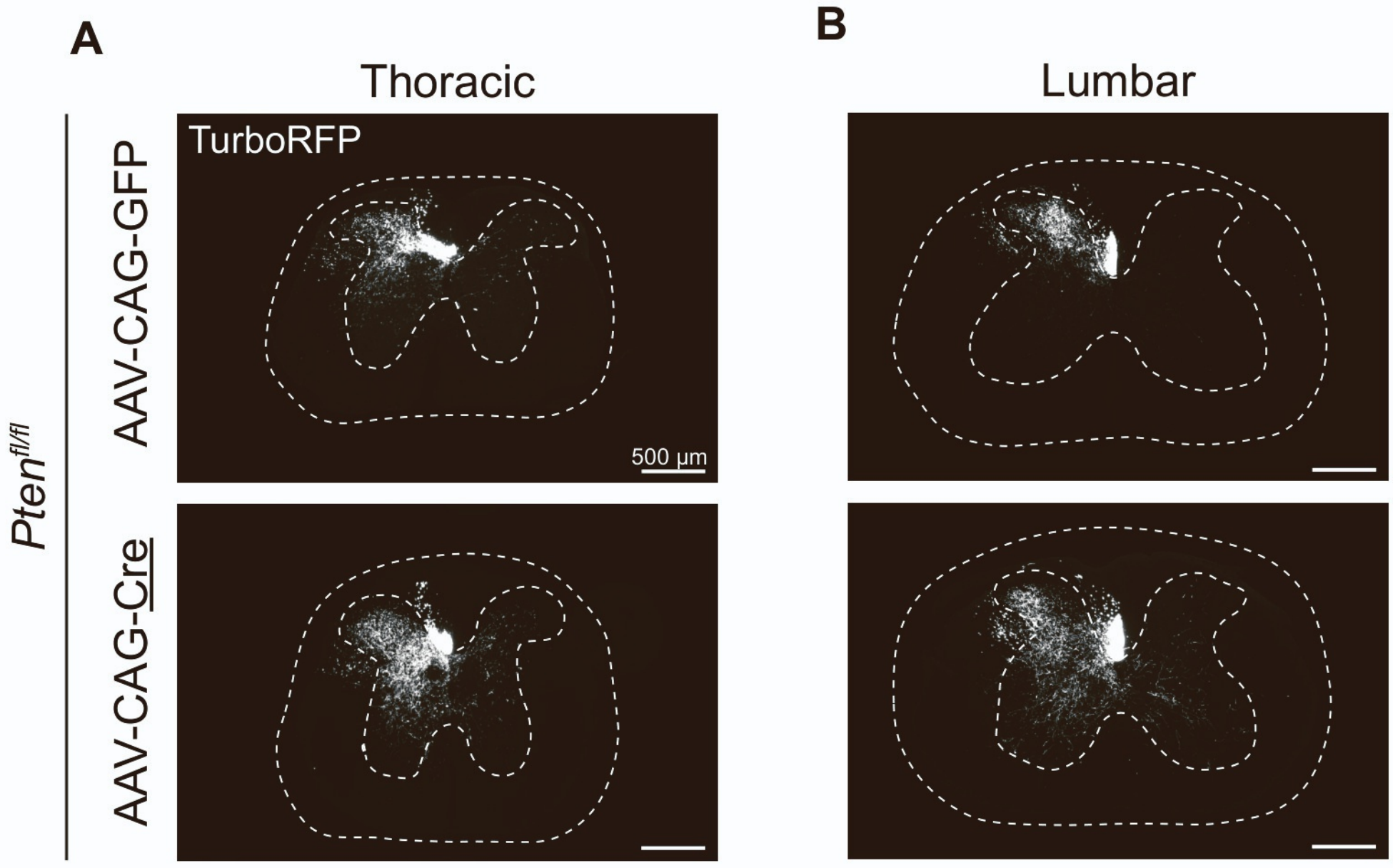

Figure S4. TurboRFP labeling of CST axons extends to thoracic and lumbar spinal cord levels. Related to Figure 4. (A) Representative transverse sections showing TurboRFP-labeled corticospinal tract axons in thoracic segments of the spinal cord from Ptenfl/fl mice injected with AAV-CAG-GFP (control) or AAV-CAG-Cre (Pten deletion) together with AAV1-hSyn-TurboRFP. **(B)** Representative transverse sections showing TurboRFP-labeled corticospinal tract axons in lumbar segments of the spinal cord from Ptenfl/fl mice injected with AAV-CAG-GFP (control) or AAV-CAG-Cre (Pten deletion) together with AAV1-hSyn-TurboRFP.

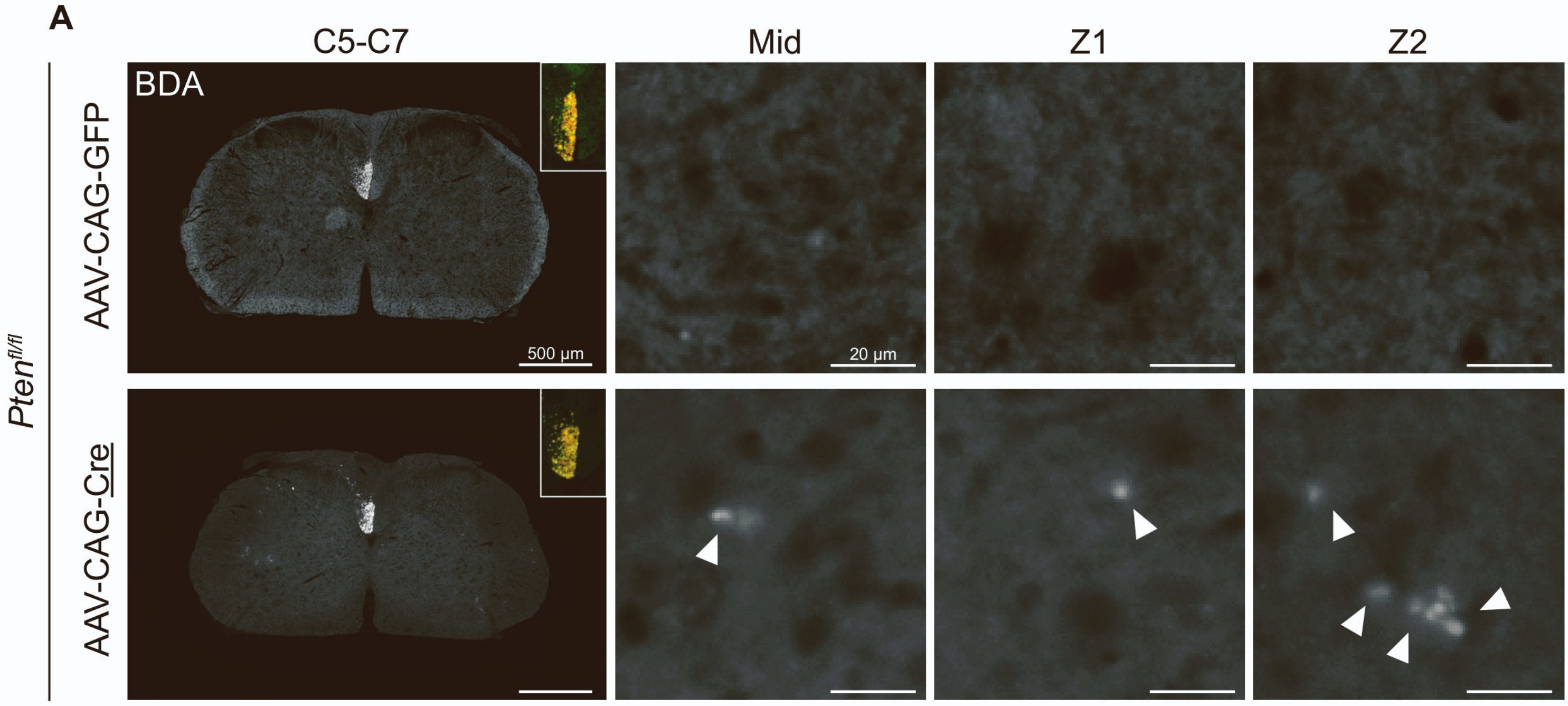

Figure S5. Detection of sparse BDA-labeled CST collaterals after unilateral pyramidotomy. Related to Figure 4. (A) Representative transverse sections of the C5–C7 cervical spinal cord from Ptenfl/fl mice injected with AAV-CAG-GFP or AAV-CAG-Cre and labeled with BDA. Higher-magnification views of the Mid, Z1, and Z2 regions illustrate the sparse and minimally detectable BDA-positive CST collateral fibers (arrowheads) observed after Pten deletion. BDA was detected using fluorescently conjugated streptavidin without signal amplification. Color insets show BDA labeling (green) and native TurboRFP signal (red) within the main CST.

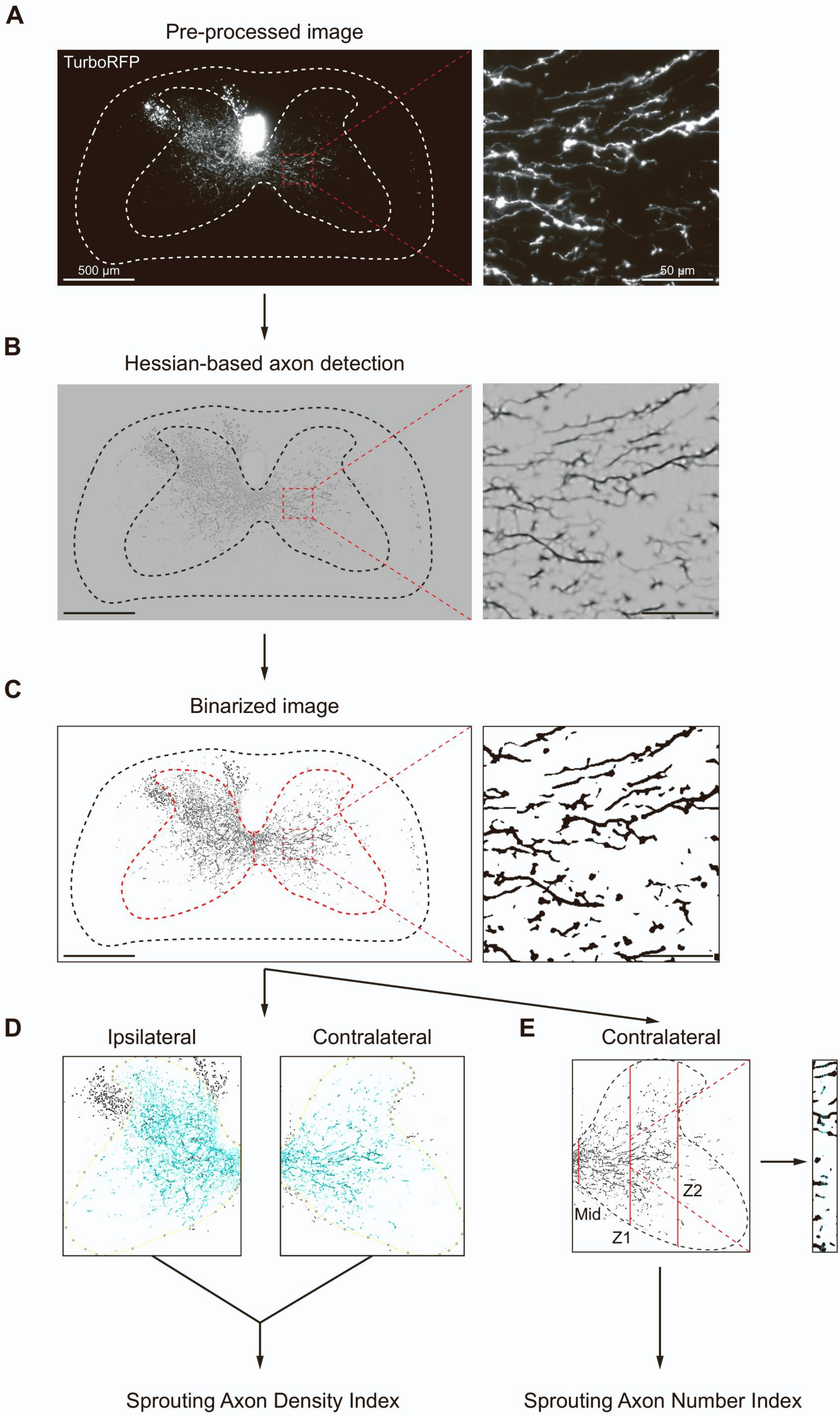

Figure S6. Semi-automated FeatureJ-based pipeline for CST sprouting analysis of CST sprouting using FeatureJ. Related to Figure 4. (A) Pre-processed TurboRFP image of a transverse cervical spinal cord section showing collateral and sprouting fibers. **(B)** Image resulting after application of smallest Hessian value, with a smoothing scale of 2, with FeatureJ plugin. **(C)** Binarized image used for subsequent analysis. **(D)** Corresponding binarized sections of the ipsilateral and contralateral gray matter used to calculate the Sprouting Axon Density Index. **(E)** Definition of mediolateral zones (Mid, Z1, Z2) within the contralateral gray matter for calculating the sprouting axon number index, representing the number of discrete axons crossing each zone.
